# Macroscopic Structural Transition of Nickel Dithiolate Capsule with Uniaxial Magnetic Anisotropy in Water

**DOI:** 10.1002/advs.202504967

**Published:** 2025-04-23

**Authors:** Tomoko Fujino, Mafumi Hishida, Masatoshi Ito, Toshikazu Nakamura, Mizue Asada, Naoya Kurahashi, Hisao Kiuchi, Yoshihisa Harada, Koji Harano, Rie Makiura, Kanokwan Jumtee Takeno, So Yokomori, Hiroshi Oike, Hatsumi Mori

**Affiliations:** ^1^ The Institute for Solid State Physics The University of Tokyo 5‐1‐5 Kashiwanoha Kashiwa Chiba 277–8581 Japan; ^2^ Department of Chemistry Faculty of Science Tokyo University of Science 1‐3 Kagurazaka Shinjuku Tokyo 162–8601 Japan; ^3^ Institute for Molecular Science 38 Nishigo‐Naka Myodaiji Okazaki Aichi 444–8585 Japan; ^4^ Synchrotron Radiation Collaborative Research Organization The University of Tokyo Sendai Miyagi 980–8572 Japan; ^5^ Center for Basic Research on Materials National Institute for Materials Science (NIMS) 1‐1 Namiki Tsukuba Ibaraki 305‐0044 Japan; ^6^ Research Center for Autonomous Systems Materialogy (ASMat) Institute of Integrated Research Institute of Science Tokyo 4259 Nagatsuda‐cho Midori‐ku Yokohama Kanagawa 226–8501 Japan; ^7^ Department of Materials Science Osaka Metropolitan University Gakuen‐cho Naka‐ku Sakai Osaka 599–8570 Japan; ^8^ PRESTO Japan Science and Technology Agency (JST) Kawaguchi Saitama 332‐0012 Japan

**Keywords:** electronic structure, magnetic properties, nanostructures, synthesis design, X‐ray absorption spectroscopy

## Abstract

Meeting the Internet of Things (IoT) demand for flexible organic spintronics requires dynamically flexible, “soft” organic magnetic materials. These materials should be capable of reordering their macroscopic assemblies in response to external stimuli. Unlike conventional rigid, “hard” crystalline organic paramagnets, that are typically composed of open‐shell π‐ or d/π‐conjugated planar molecules and rely on intermolecular interactions in the ordered, assembled structures, soft paramagnets necessitate a delicate balance between long‐range structural order (essential for controlling magnetic properties) and dynamic flexibility a challenge previously unmet for open‐shell planar molecules. In this study, an amphiphilic d/π‐conjugated nickel dithiolate radical anion salt is presented that self‐assembles into ordered membranes, forming capsule‐like macrostructures with exceptional stability in aqueous environments. This design achieves the desired balance. These assemblies exhibit uniaxial magnetic anisotropy driven by significant spin–spin interactions and undergo temperature‐dependent macroscopic structural transitions representing, to the knowledge, the first observation of such behavior for assemblies of open‐shell planar molecules. This well‐defined, single‐molecular‐weight system provides critical structural and mechanism insights for soft matter design and a versatile platform for spintronic applications. The findings advance the development of flexible, tunable molecular soft paramagnets, expanding their potential for innovative applications in flexible devices and beyond.

## Graphical abstract

An amphiphilic open‐shell d/π‐conjugated nickel dithiolate salt forms a bilayer capsule exhibiting uniaxial magnetic anisotropy, driven by spin–spin interactions. The capsule, exceptionally stable in aqueous environments, undergoes temperature‐dependent structural transitions representing, to the knowledge, the first observation of such behavior for assemblies of open‐shell planar molecules. This design balances long‐range order and flexibility, addressing constraints of static organic paramagnets.

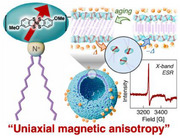

## 1 Introduction

**Figure 1:**
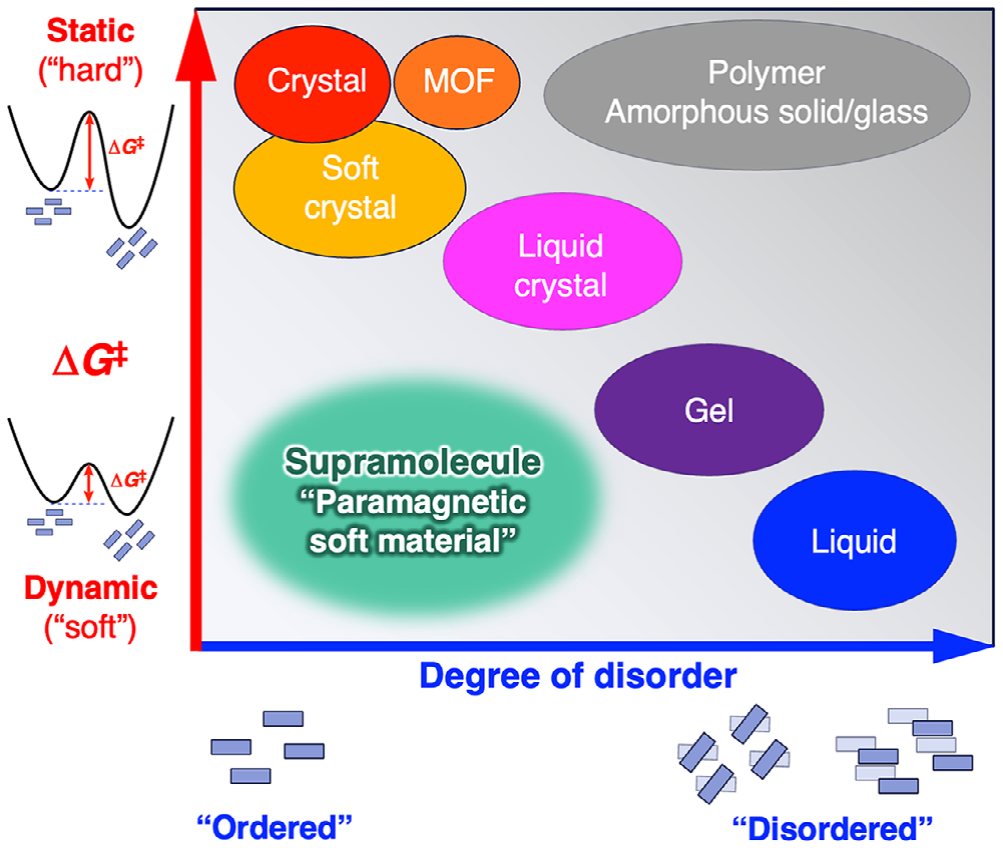
Relationship between degree of disorder and activation energy (Δ*G* ^‡^) required for dynamics for open‐shell planar. This figure illustrates the general trade‐off between the degree of disorder in materials and Δ*G* ^‡^ required for transitions to other states (Adapted from the ref. [7]). Supramolecular materials are an exception to this trend, exhibiting a high degree of order and dynamic behavior. This unique combination makes them promising candidates for the development of paramagnetic soft materials, as demonstrated in this study.

Organic magnetic materials are integral to spintronics research, leveraging tunable spin–spin interactions to advance fundamental understanding and applications.^[^ ^1^, ^2^, ^3^ ^]^ The growing demand for dynamically flexible (i.e., “soft”) organic paramagnetic materials, driven by the rapid expansion of the Internet of Things (IoT)—particularly in spintronic wearable devices^[^ ^4^, ^5^ ^]^—presents both opportunities and challenges. Conventional rigid (i.e., “hard”) molecular‐based paramagnetic materials in spintronic research often consist of π‐ or d/π‐conjugated planar molecules and rely on effective intermolecular interactions within their assembled, stacked forms. In contrast, soft organic paramagnets, capable of adapting their assembled structure in response to external stimuli, promise advanced functionalities for a wide range of applications,^[^ ^6^ ^]^ from magnetically guided drug delivery to stimuli‐responsive devices.^[^ ^7^, ^8^, ^9^, ^10^, ^11^, ^12^, ^13^, ^14^, ^15^, ^16^, ^17^, ^18^, ^19^, ^20^ ^]^ However, realizing this potential requires precise control over spin–spin interactions within a flexible and dynamic framework, a feat previously unachieved with open‐shell planar molecules. Reaching this potential necessitates balancing the long‐range structural order (essential for controlled magnetic properties) with the inherent flexibility of soft matter. Increased structural order often enhances spin–spin interactions, but it typically compromises the flexibility required for dynamic soft materials. This trade‐off reflects an inverse relationship between the structural order and the activation energy barrier (Δ*G* ^‡^) associated with the structural state changes (**Figure** **1**).^[^ ^7^ ^]^ Rigid materials with high Δ*G* ^‡^ values are typically structurally ordered, as observed in typical organic paramagnets,^[^ ^2^, ^3^ ^]^ whereas highly dynamic soft materials with low Δ*G* ^‡^ often lack sufficient structural order. Overcoming this trade‐off necessitates design principles that enable open‐shell planar molecules to integrate into assemblies with both dynamic and ordered features to control spin–spin interactions.

Current strategies for creating soft magnetic materials—including incorporating magnetic nanoparticles into films,^[^ ^8^, ^9^, ^10^, ^11^, ^12^, ^13^, ^14^ ^]^ encapsulating them within soft assemblies,^[^ ^15^, ^16^, ^17^ ^]^ or embedding magnetic components into surfactants^[^ ^18^, ^19^, ^20^, ^21^ ^]^—often face critical limitations. A key challenge is structural heterogeneity, such as uneven nanoparticle distributions, which impedes precise magnetic manipulation of spin–spin coupling.^[^ ^9^, ^13^, ^18^ ^]^ This heterogeneity also hinders the investigation of structure–property relationships and obscures the mechanisms underlying stimuli‐responsive behavior. Thus, open‐shell single‐molecular‐weight systems that balance long‐range order with dynamic flexibility are crucial for elucidating these mechanisms, advancing soft organic paramagnet design, and enabling next‐generation spintronic materials. Open‐shell planar molecules, in particular, are readily stacked to form highly ordered assemblies with anisotropic intermolecular interactions, exhibiting unique and controllable magnetic properties.

Amphiphilic assemblies^[^ ^22^, ^23^, ^24^, ^25^, ^26^, ^27^ ^]^ offer a promising supramolecular approach^[^ ^28^, ^29^, ^30^, ^31^, ^32^ ^]^ to reconcile competing requirements of long‐range order and flexibility. These systems form dynamically flexible membrane structures in water that undergo temperature‐, pH‐, or additive‐induced macrostructural transitions.^[^ ^22^, ^23^, ^24^, ^25^, ^26^, ^27^ ^]^ However, integrating spin‐active, open‐shell components into such assemblies poses significant challenges due to their chemical instability and susceptibility to degradation in aqueous and oxidative environments. Open‐shell planar species, such as radical anions or cations, are particularly sensitive, requiring stabilization through strong orbital overlap and intermolecular interactions.^[^ ^2^, ^3^ ^]^

**Figure 2:**
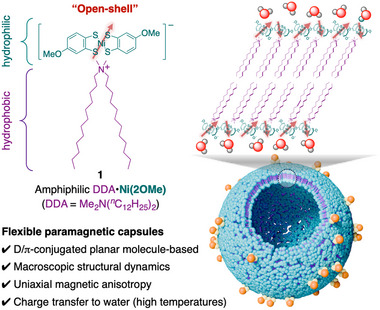
The self‐assembly of an open‐shell, planar, and amphiphilic nickel **Ni(2OMe)** anion salt **1** in water, forming dynamically flexible paramagnetic capsule, is demonstrated in this study. The red arrow indicates a π‐spin delocalized over the anion molecule, including Ni atom.

In this study, we extend a supramolecular approach to assemblies of open‐shell planar molecules by designing an amphiphilic d/π‐conjugated planar nickel dithiolate. The nickel dithiolate radical anion acts as a counter anion to the hydrophobic alkyl chains, forming a tail structure^[^ ^33^ ^]^ (**Figure** **2**). The anion salts self‐assemble into membranes that balance long‐range order and dynamic flexibility, exhibiting exceptional stability in aqueous environments. Unlike previous amphiphilic nickel dithiolate salts, which were partially incorporated into vesicular films and lacked the effective intermolecular interactions necessary for efficient spin–spin coupling,^[^ ^34^ ^]^ our designed open‐shell planar building blocks readily stack and self‐assemble into membranes that exhibit uniaxial magnetic anisotropy via significant intermolecular spin–spin interactions. The assemblies form capsule‐like macroscopic structures and undergo temperature‐dependent structural transitions—representing, to our knowledge, the first observation of such behavior for assemblies of open‐shell planar molecules. Our findings provide valuable insights for the development of advanced paramagnetic materials with tunable properties, broadening their potential applications in nanomedicine and beyond.

## 2 Results and Discussion

### 2.1 Molecular Design and Synthesis of Amphiphilic Salt

**Scheme 1:**
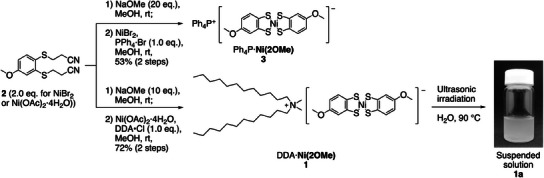
Synthesis of Ni(2OMe) anion salts 4 and 1, and the assembly of 1 in water (1a).

**Figure 3:**
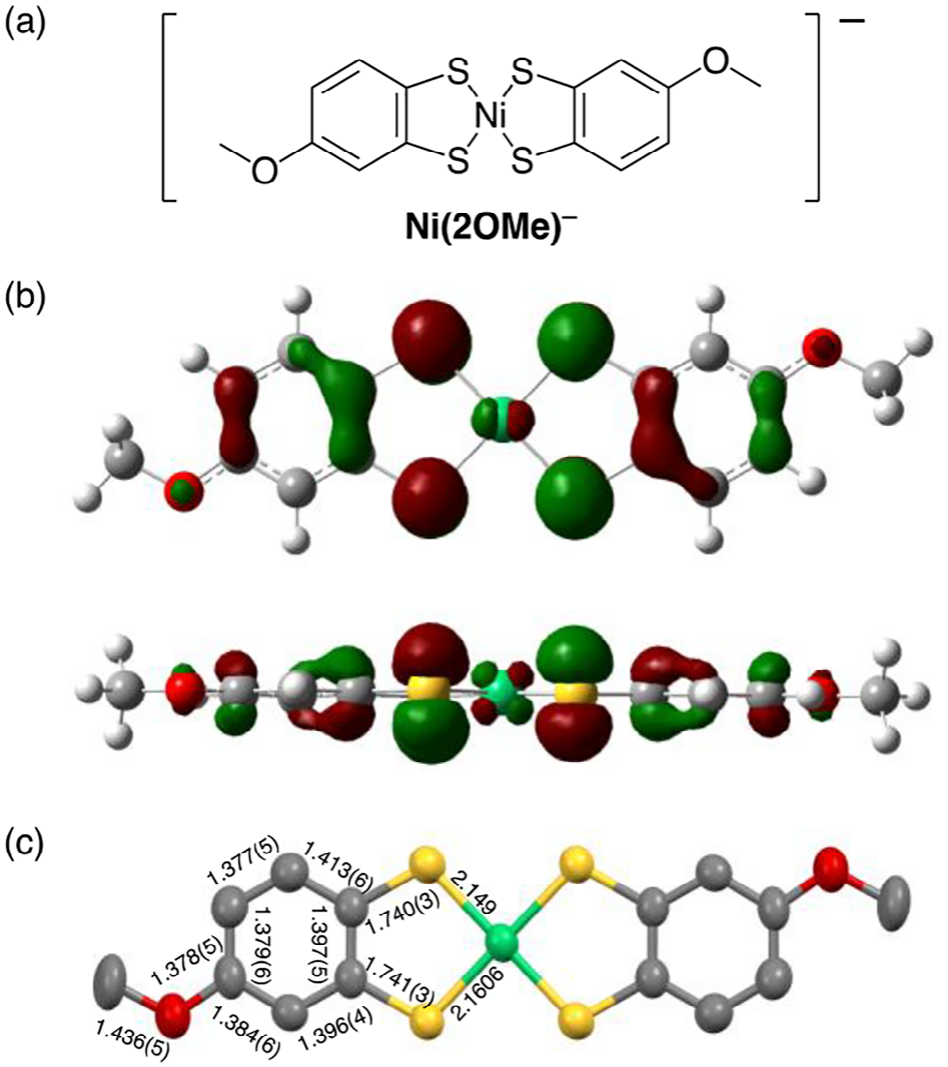
Structure of the **Ni(2OMe)** anion in *trans* configuration. a) Chemical structure. b) Singly occupied molecular orbital (SOMO) calculated using Gaussian software (UB3LYP/6‐311G++(d,p), SDD for Ni). The orbital delocalized over the anion, including Ni atom. (Top) The top view. (Bottom) The side view. c) Single‐crystal structure of anion salt **4** with different bond lengths. Hydrogen atoms are omitted for clarity. Atoms are colored as follows. Nickel: green, carbon: gray, sulfur: yellow, oxygen: red, hydrogen: white.

Initially, we designed and synthesized an amphiphilic salt by combining a bis(4‐methoxybenzene‐1,2‐dithiolato)nickelate(III) anion and an ammonium cation containing long hydrophobic alkyl chains derived from ligand precursor **2**.^[^ ^35^ ^]^ This was achieved through decyaoethylation of **2** under basic conditions, followed by coupling with nickel(II) diacetate tetrahydrate^[^ ^36^ ^]^ in the presence of didodecyldimethylammonium (DDA)^[^ ^37^ ^]^ chloride, yielding DDA·**Ni(2OMe)** (compound **1**, **Scheme** **1**). To prepare a reference compound, a reaction with tetraphenylphosphonium bromide followed by transmetallation produced Ph_4_P·**Ni(2OMe)** (compound **4**). These synthetic procedures are scalable, enabling the production of hundreds of milligrams in a single sequence. The structural integrity of the **Ni(2OMe)** anion is confirmed by analyzing its single‐crystal structure of **4** (Figure S1; Table S1, Supporting Information).^[^ ^38^ ^]^ The single crystal consisted of a 1:1 ratio of cations to anions, confirming the monoanionic nature of the **Ni(2OMe)** anion with a square‐planar Ni center. The space group of **4** was identified as *P*–1, confirming two crystallographically half‐molecule‐independent anions in the unit cell: one ordered and the other orientationally disordered in a 63:37 ratio. The bulky cations in the single‐crystal structure likely hinder effective intermolecular interactions between the anions, resulting in the electronic structure resembling that of the isolated anion. The two ligands in the **Ni(2OMe)** anion adopt a *trans* configuration (**Figure** **3**; Figure S1, Supporting Information). Bond‐length analysis of the ligand (Figure 3c; Figure S1b, Supporting Information) reveals two distinct Ni–C bond lengths in the ordered anion (2.1606 and 2.149 Å; Figure 3c), indicating intramolecular polarization caused by the electron‐donating 4‐methoxy group in the ligand. The electronic asymmetry of the ligand is further supported by the distorted shape of the singly occupied molecular orbital (SOMO) along the short‐axis of the molecule, as revealed by the density functional theory (DFT)‐optimized structure using the Gaussian software (UB3LYP/6‐311G++(d,p), SDD for nickel; Figure 3b; Table S3, Supporting Information). The planar structure, devoid of bulky substituents observed in the single‐crystal and DFT‐optimized structures, suggests the potential for efficient stacking assemblies with favorable intermolecular spin–spin interactions. Furthermore, the SOMO is primarily delocalized over the anion molecule, including the Ni atom, which may contribute to the enhanced aqueous stability of the open‐shell species.

### 2.2 Assembly of Amphiphilic Nickel Dithiolate Salt in Water

**Figure 4:**
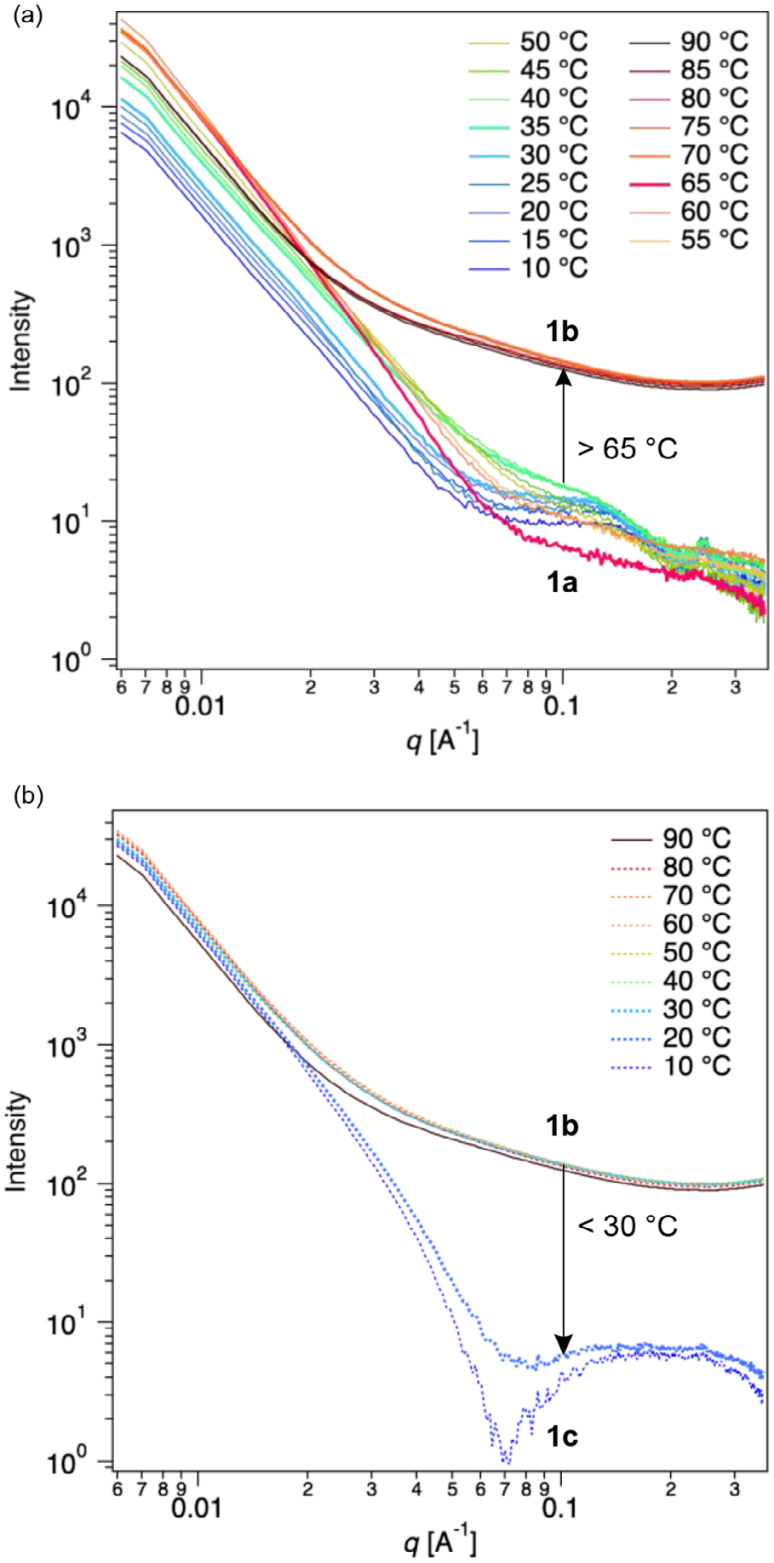
Temperature‐dependent small‐angle X‐ray scattering data of **1a** during the heating (a) and cooling (b) processes. Assembly forms change from **1a** to **1b** during the heating process and from **1b** to **1c** during the cooling process in a hysteretic manner.

The amphiphilic nickel dithiolate salt **1** was next dispersed in water using ultrasonication. A mixture of **1** and water was subjected to ultrasonic irradiation at 90 °C, followed by slow cooling over 12 h, resulting in a whitish‐green suspension **1a** (≈1 µm; Scheme 1). The green color is a characteristic feature of nickel bisdithiolenes and is mainly attributed to the SOMO‐to‐LUMO (lowest unoccupied molecular orbital) transition (Table S2, Supporting Information). The transition arises from hybridized orbitals formed between nickel ions and the ligands. The whitish appearance indicates its dispersed state in water. The dispersion was then analyzed by small‐angle X‐ray scattering (SAXS) with the scattering vector *q* range, revealing the absence of diffraction peaks. This data indicates the absence of a multilamellar structure, whose structure may be affected by the low concentration. The scattering intensity was dominated by the bilayer form factor. The primary peak at the lowest *q* corresponds to the characteristic first hump of the bilayer form factor, with subsequent oscillatory scattering patterns (**Figure** **4**a). A dip location in the scattering intensity ≈ 0.1 Å^−1^ is consistent with the thickness of a bilayer membrane, as previously reported.^[^ ^37^ ^]^

**Figure 5:**
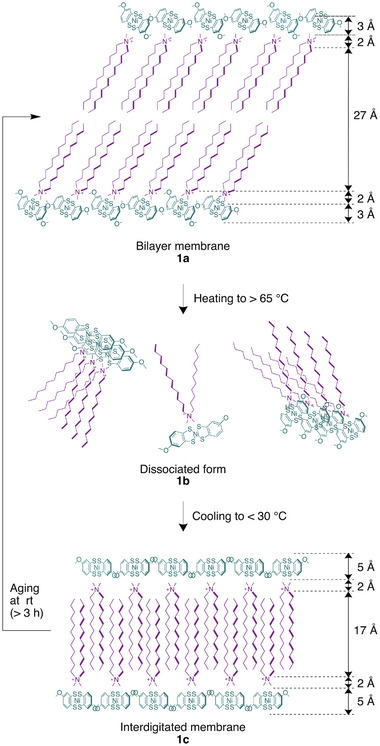
Possible temperature‐dependent structural changes: bilayer membrane **1a**, dissociated form **1b**, and interdigitated membrane **1c**.

The bilayer thickness was quantified by analyzing the electron density (ED) value^[^ ^39^ ^]^ obtained from the SAXS profiles (Figure S2, Supporting Information), assuming a vesicle model with the hydrophobic alkyl chains oriented parallel to the bilayer planes. The analyses revealed a membrane structure with an inner hydrophobic layer thickness of 27 Å, comprising the alkyl chains. This thickness exceeds the individual alkyl chain lengths (16 Å)^[^ ^40^ ^]^ but is shorter than twice the chain length, indicating the bilayer is formed by an amphiphilic salt **1** with alkyl chains inclined out‐of‐plane (**Figure** **5**, top). Conversely, the outer hydrophilic head region of the membrane, comprising the **Ni(2OMe)** anion, exhibited a thickness of 3 Å. Considering the short‐axis length of the anion of ≈5 Å,^[^ ^41^ ^]^ this shorter thickness (3 Å) suggests that the anions are stacked while inclining along the long‐axis and shifting along the short‐axis of the molecule (Figure 5, top). Wide‐angle X‐ray scattering (WAXS) profiles of **1a** showed comparatively broad diffraction peak, seemingly reflecting intermolecular interactions. This suggest that the structure forms a highly ordered phase (likely a gel phase), although not a periodic crystalline phase. The peaks at *q* of 1.5 and 1.6 Å^−1^ (Figure S5, Supporting Information) correspond to stacking distances of 4.2 and 3.9 Å, respectively. This observation suggests the in‐plane ordering of the DDA alkyl chain^[^ ^26^ ^]^ and stacking of the nickel dithiolate anions. Compared to the π–π stacking distance of ≈3.5 Å observed in the single‐crystal nickel dithiolene complex,^[^ ^36^ ^]^ the slightly increased intracolumnar stacking distance (3.9 Å) indicates that either a shift of anion molecules along the short‐axis or the interference from the long alkyl chains of the counter cations hinder the efficient π–π stacking. Notably, the membranes exhibited exceptional stability in water for several months. This stability is primarily attributed to their stacking structure to promote intermolecular spin–spin interactions (vide infra) or the delocalization of the SOMO over the anion molecule.

### 2.3 Structural Transition of Nickel Dithiolate Membrane

The bilayer membranes exhibited unique temperature‐dependent structural transitions. When heated above 65 °C, the SAXS profiles showed substantial changes with the disappearance of significant form factors (Figure 4a), indicating membrane dissociation (**1b** in Figure 5, middle). Such a dynamic structural change was previously unachieved in open‐shell planar molecule‐based paramagnets. Considering the *q* dependence at the low region at high temperatures, the dissociated form **1b** likely disassembles into smaller assemblies, resulting in a mixture of coexisting structures such as membranes, micelles, and monomers. Notably, this structural transition was reversible but exhibited hysteresis; the dissociated form **1b** persisted until the temperature dropped to 30 °C. Upon further cooling below 30 °C, a SAXS profile corresponding to **1c** appeared (Figure 4b). Intriguingly, after aging the solution at room temperature for more than 3 h, the SAXS profile corresponding to **1a** is recovered (Figure S4, Supporting Information). This phenomenon suggests that **1c** is a metastable state under kinetic control,^[^ ^42^ ^]^ where the dynamic transition depends on the cooling rates.^[^ ^2^ ^]^ These results demonstrated that the assembly of **1** in water uniquely exhibited reversible but hysteretic dynamic changes in the membrane structures, exhibiting transitions among the three states: from the thermodynamically stable bilayer membrane **1a**, through dissociated form **1b**, to the metastable membrane **1c**. This hysteretic dynamic change is a rare feature among typical aggregates constituting amphiphilic molecules.^[^ ^26^, ^37^ ^]^ The observed metastable state (**1c**) may be induced by spin–spin intermolecular interactions between planar molecules (vide infra) or kinetically favored antiparallel orientation of alkyl chains, contributing to the stabilization of **1c** during the transition process.

The radial ED distribution analysis^[^ ^39^ ^]^ revealed that the hydrophobic alkyl chain moiety in **1c** was 17 Å thick, shorter than that in state **1a**. This thickness was nearly equivalent to that of a single alkyl chain (16 Å),^[^ ^40^ ^]^ suggesting an interdigitated membrane structure (Figure 5, bottom).^[^ ^26^ ^]^ In contrast, the thickness of the hydrophilic **Ni(2OMe)** anion moiety increased to 5 Å, which was comparable to the short‐axis molecular length (≈5 Å).^[^ ^36^, ^41^ ^]^ This observation indicates intracolumnar stacking without significant shifts along the short‐axis of the molecule. The WAXS profiles exhibited scattering exclusively at *q* = 1.5 Å^−1^ (i.e., 4.2 Å; Figure S5, Supporting Information), likely attributed to the in‐plane alkyl chain ordering, with no significant scattering at 1.6 Å^−1^. These results suggest that the elongation of the anion–anion distances in interdigitated membrane **1c** may weaken the anion‐stacking interaction.

### 2.4 Macroscopic Structural Transition in the Membrane

**Figure 6:**
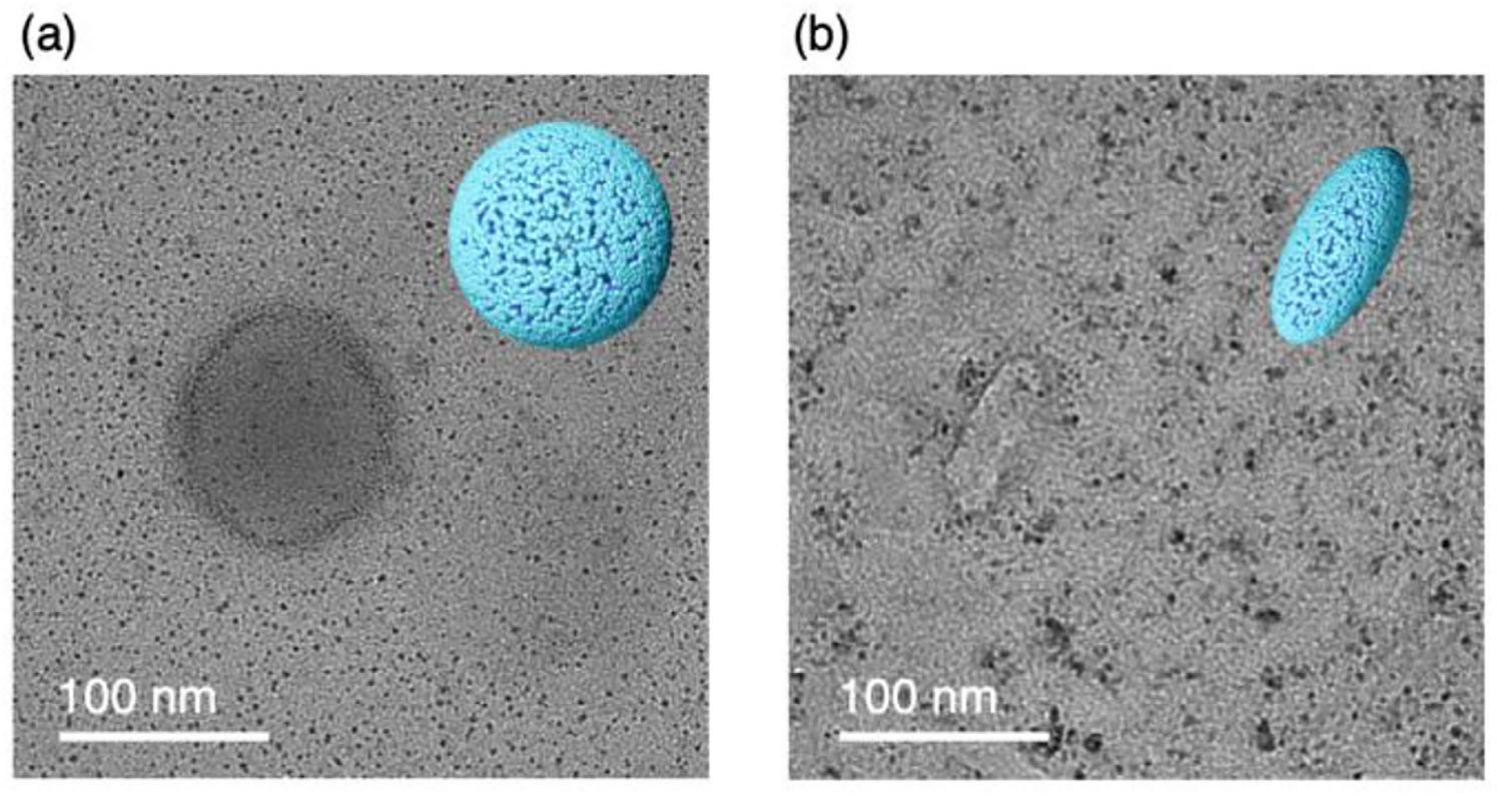
Transmission electron microscopy (TEM) images of dried samples of **1a** (a) and **1c** (b) on films supported by a TEM microgrid. Other images are shown in Figure S6 (Supporting Information). Illustrations of the assembly forms are shown in the images.

The structural details of molecular assembly of **1** within membranes provided insights into the macroscopic structures and their temperature‐dependent transitions. Transmission electron microscopy (TEM) observation of bilayer membrane **1a** revealed circular capsule structures with diameters ranging from 50 to 100 nm (**Figure** **6**a; Figure S6a, Supporting Information), consistent with the dynamic light scattering (DLS) analysis, which estimates the average size of **1a** to be approximately 55 nm (Figure S7a, Supporting Information). These TEM images closely resemble the bilayer vesicles formed in water that collapsed under the vacuum conditions of TEM observation.^[^ ^43^ ^]^ The bilayer membrane structure indicated by the ED analyses of **1a** (Figure S2, Supporting Information) further supports the formation of spherical bilayer vesicle‐like capsules in water. Notably, the macroscopic structures were observed without staining the TEM sample with metallic reagents, confirming the presence of nickel atoms in the assemblies. In contrast, the TEM image of **1c** exhibited a flat, elliptical shape (Figure 6b; Figure S6b, Supporting Information), with sizes ranging from 50 to 100 nm, similar to **1a**, suggesting the formation of an ellipsoidal vesicular structure in water. Despite the difference in macroscopic shapes, the average size of **1c** is also estimated to be approximately 55 nm by the DLS analysis (Figure S7b, Supporting Information), consistent with **1a**. The membrane structural changes from the bilayer membrane (**1a**) to interdigitated membrane (**1c**) may result in the macroscopic structural changes observed in TEM images. Notably, the DLS data at high temperatures indicates the average size of **1b** is estimated to be 25 nm. This supports the conclusion that the dissociated state **1b** comprises a mixture of coexisting structures, such as membranes, micelles, and monomers. This is also supported by SAXS data in the low *q* range (Figure 4; middle in Figure 5) and suggests that **1b** is not the completely soluble in water. The dissociated state **1b** in water may enhance the chemical stability of the open‐shell molecule **1** by maintaining the effective intermolecular interactions.

### 2.5 Intermolecular Spin–Spin Interaction in Bilayer Capsules

**Figure 7:**
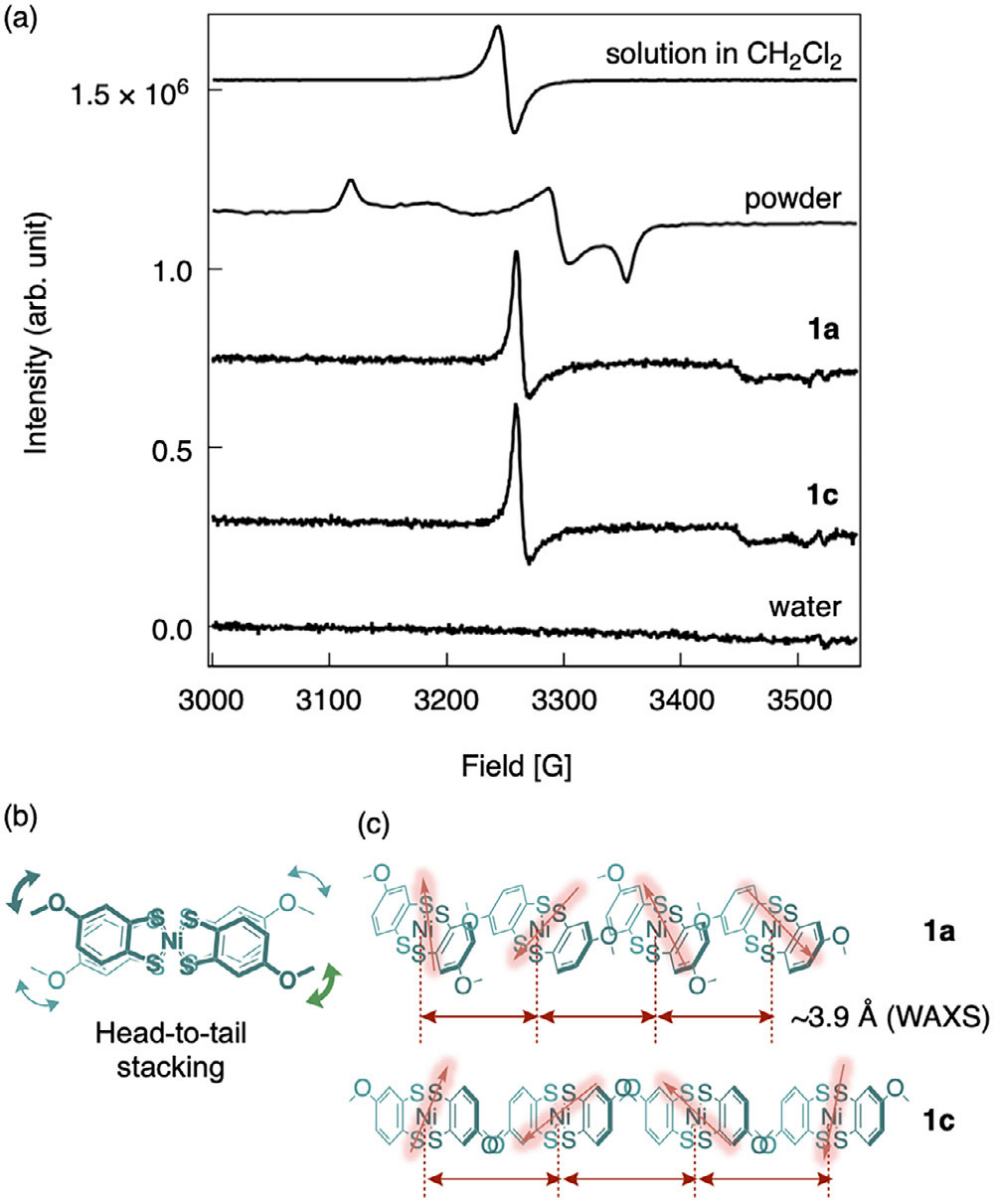
a) Electronic spin resonance spectra (ESR) of the **Ni(2OMe)** anion salt in dichloromethane, powder, **1a**, and **1c**, with water as a background. b) Possible stacking in a head‐to‐tail manner. c) Possible orientation of **Ni(2OMe)** anions in the bilayer membrane (**1a**) and interdigitated membrane (**1c**).

The bilayer capsules **1a** and **1c** exhibited unique magnetic properties in water, distinct from those in their isolated states. Their electronic spin resonance (ESR) spectra demonstrated unique paramagnetic behavior with uniaxial anisotropy (**Figure** **7**a; Figure S8, Supporting Information), which significantly differed from those observed in the solution and powder forms. In dichloromethane, a single Lorentzian curve was observed (representing the isolated state) due to Brownian motion. In contrast, the powders (polycrystals) displayed a typical triaxial anisotropy spectrum, consistent with a monoanionic square‐planar Ni(III) dithiolate anion possessing a rhombic *g* tensor.^[^ ^44^ ^]^

The magnetic anisotropy, observed for **1a** and **1c**, has previously been observed only in highly long‐range ordered (crystalline) conductive polymer films^[^ ^45^ ^]^ and a limited number of single crystals with strong spin–spin interactions.^[^ ^46^, ^47^, ^48^ ^]^ This observation supports the presence of long‐range order in **1a** and **1c**. Remarkably, this observation of paramagnetic behavior with uniaxial anisotropy, driven by strong intracolumnar spin–spin interactions, is unprecedented among soft organic magnetic materials. This behavior is likely due to the planar structure of the **Ni(2OMe)** anions and their specific orientation within the macroscopic assemblies (Figure 5). The 4‐methoxy groups in the ligands appear to induce a head‐to‐tail stacking arrangement of the **Ni(2OMe)** anions, minimizing steric hindrance from the steric 4‐methoxy substituents. This stacking configuration facilitates molecular fluctuations via rotation along the stacking direction (Figure 7b,c). This fluctuation may impede the formation of a periodic crystalline phase, potentially leading to a highly ordered phase (likely a gel phase), as indicated by WAXS data. Additionally, the SOMO of the **Ni(2OMe)** anion, characterized by its nodal structure along the molecular long‐axis (Figure 3b), supports intracolumnar orbital overlap in the head‐to‐tail arrangement, even with slight uniaxial rotating fluctuations.^[^ ^49^ ^]^

The uniaxially anisotropic electronic structure observed in both **1a** and **1c** highlights significant intracolumnar spin–spin interactions shared by these capsules. The potential uniform columnar stacking of the planar **Ni(2OMe)** anions in **1a** and **1c** likely generates a consistent electronic environment of d/π‐spins, resulting in identical uniaxial magnetism in both forms. Although the WAXS data (Figure S5, Supporting Information) suggests that the intracolumnar stacking distance in **1c** may be longer than that in **1a**, the uniform stacking appears to mitigate the impact of this difference (Figure 7c), thereby maintaining robust magnetic properties. Moreover, the strong intracolumnar spin–spin interactions likely contribute to the exceptional stability of the capsules in water, which is sustained over several months. Magnetic anisotropy in soft organic materials heavily relies on spin–spin interactions, especially in systems composed of light elements. The strong anisotropic spin–spin interactions observed in this study suggest a promising design strategy for creating soft organic paramagnets with stable and tunable properties.

Their absorption spectra further confirmed the comparable electronic structures of capsules **1a** and **1c**. At 20 °C, the UV spectra of both forms exhibit similar absorption peaks at 902 and 800 nm (Figure S9, Supporting Information), possibly corresponding to SOMO‐to‐LUMO and HOMO–*n*‐to‐LUMO transitions, respectively. These transitions were simulated by TDDFT calculations (Gaussian software, UB3LYP/6‐311G++ (d,p), SDD for Ni; Table S2, Supporting Information). Upon heating the sample above 65 °C, the dispersed form **1** was generated, accompanied by the appearance of a new absorption band at 960 nm, which was absent in the spectra at low temperatures. This observation suggests the emergence of a unique electronic structure at high temperatures in water, likely attributed to the intramolecular charge transfer between the metal and ligands. The intramolecular charge transfer may be facilitated by hydrogen bonding with the surrounding water molecules,^[^ ^35^ ^]^ as supported by TDDFT simulations (Figures S10 and S11; Tables S4–S7, Supporting Information). The energy level shifting may be induced by the HOMO−*n* to LUMO transition in the presence of a hydrogen‐bonded water molecule (Table S2, Supporting Information).

### 2.6 Electronic Structure in Nickel Atom within the Capsules

**Figure 8:**
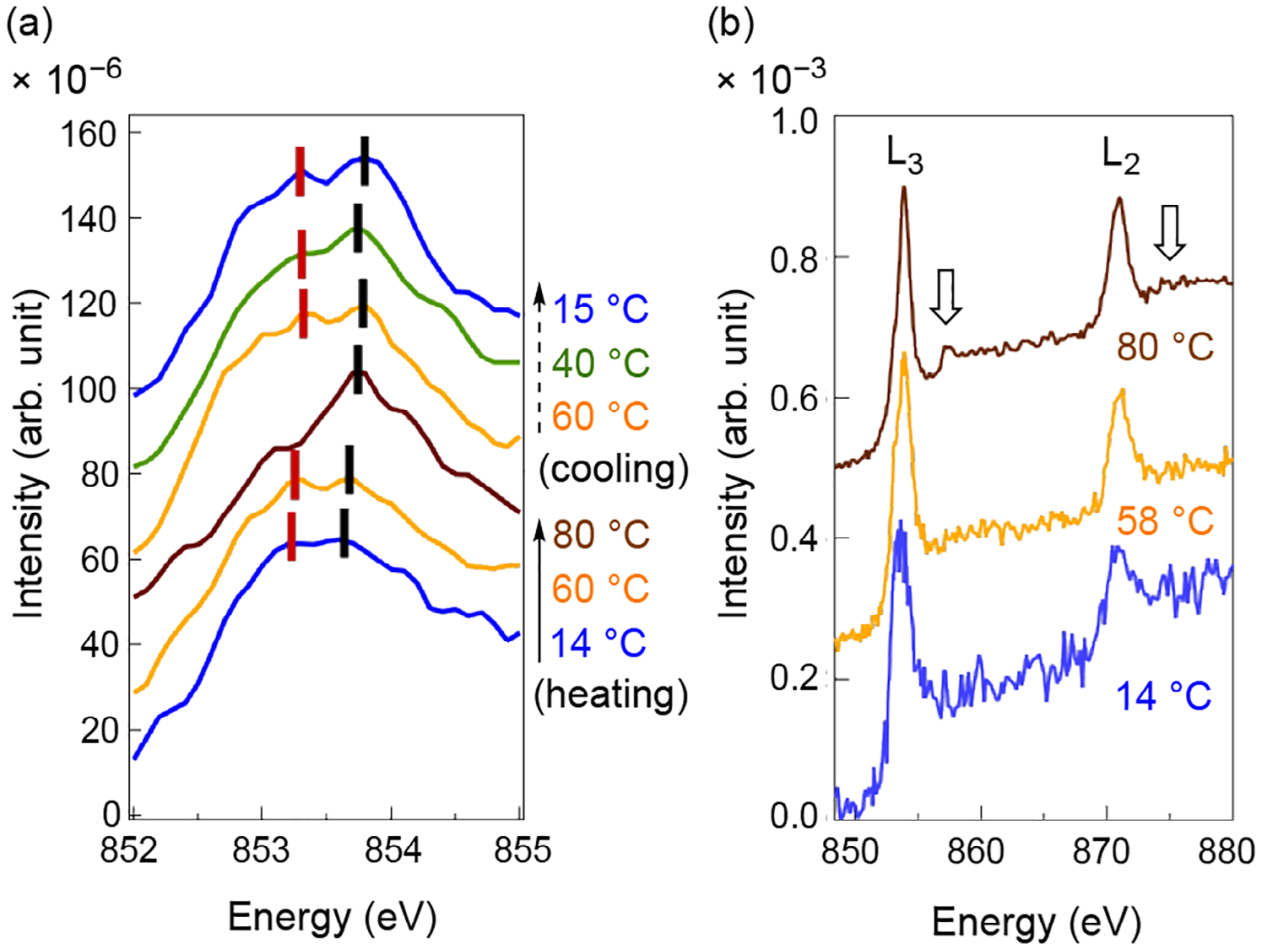
Ni X‐ray absorption spectroscopy (XAS) for Ni atoms of the **Ni(2OMe)** anion salt in capsule **1a**. a) Spectra of the Ni L_3_ region during the heating and cooling processes. b) Spectra in the broad ranges covering Ni L_3_ and L_2_ during the heating and cooling processes, with satellite signals indicated by arrows.

Insights into the unique dynamic electronic structures of the bilayer capsules **1a** and **1c** and their temperature‐dependent behavior in water were obtained through X‐ray absorption spectroscopy (XAS).^[^ ^50^, ^51^ ^]^ The Ni L‐edge XAS spectrum of **1a** at 14 °C exhibits two major peaks at 853.2 and 853.7 eV (**Figure** **8**), corresponding to the L_3_ absorption region edge of nickel atoms. The broad spectral features likely reflect band dispersion arising from intermolecular interactions between the **Ni(2OMe)** anions in water. These finding supports the spin–spin interactions, as suggested by the ESR spectra results (Figure 7; Figure S8, Supporting Information), with the SOMO delocalized over the anion molecule and including Ni atom. The spectral shapes remained consistent within the ranges of 14–60 °C, aligning with the SAXS profiles (Figure 4a). However, at 80 °C, the membrane structure **1a** disassembled to smaller assemblies in the dissociated form **1b**, and the absorption transformed into a sharper peak centered at 853.7 eV, indicating a change in the electronic structure of the Ni atom within the **Ni(2OMe)** anion. This change was likely attributed to the disruption of intermolecular Ni–Ni interactions of the **Ni(2OMe)** anions that were present at 60 °C and below.

Notably, the XAS spectrum at higher energy ranges revealed the emergence of charge‐transfer satellite peaks at 80 °C, appearing on the long‐wavelength side of the L_3_ and L_2_ absorption edges (Figure 8b). These observations underscore significant alterations in the electronic structures of the Ni atom, driven by temperature‐induced structural transition in the capsule. The satellite peaks likely arise from changes in charge transfer between the Ni atom and ligands,^[^ ^52^ ^]^ influenced by the surrounding water molecules and possibly facilitated by the hydrogen bonding between ligands and water molecules.^[^ ^35^ ^]^ This unique phenomenon is supported by the observed changes in the absorption spectrum (Figure S9, Supporting Information) and corresponding simulations (Figures S10 and S11; Table S2, Supporting Information). The temperature‐dependent dynamic structural change enables alterations in the electronic structure of the **Ni(2OMe)** anion through both intermolecular and intramolecular interactions, involving the surrounding water molecules. These findings highlight the unique properties of the d/π‐conjugated planar molecule‐based soft paramagnet capsule in water. The dynamic microscopic and macroscopic geometric and electronic structural changes, potentially involving hydrogen‐bonding with surrounded water molecules, suggest the potential for advanced applications in soft magnetic materials.

## 3 Conclusion

We addressed the trade‐off between achieving long‐range structural order and preserving the inherent dynamic flexibility in soft materials composed of open‐shell planar molecules a common challenge in molecular paramagnets by developing an amphiphilic assembly of the hydrophilic d/π‐conjugated **Ni(2OMe)** anion. The resulting bilayer membrane structure in water exhibited a unique arrangement, with anions stacking and shifting along the short‐axis of the molecules to form spherical capsules. The assembly exhibited temperature‐dependent hysteretic transformations, transitioning from the bilayer membrane (**1a**) to a dissociated form (**1b**) and subsequently to an interdigitated membrane (**1c**), which likely adopted ellipsoidal structures. Effective intermolecular spin–spin interactions between the open‐shell d/π‐conjugated **Ni(2OMe)** anion molecules contributed to the formation of a band‐like structure, enabling dynamic electronic functionalities. These interactions were modulated by the rotational fluctuation of the anions along the stacking direction, influenced by the molecular design features such as the hydrophilic 4‐methoxy group. This fluctuation imparted uniaxial magnetic anisotropy to both **1a** and **1c**. At elevated temperatures above 65 °C, the assembly dissociated, disrupting significant Ni–Ni interactions, as evidenced by the narrowing Ni XAS signals. The emergence of satellite features in the L_3_ and L_2_ bands of the Ni XAS spectrum and long‐wavelength UV absorption suggested that intramolecular charge transfer between the metal and ligands^[^ ^52^ ^]^ might occur, likely facilitated by hydrogen bonding with surrounding water molecules.^[^ ^35^ ^]^ These macroscopic dynamic structural and electronic changes were previously unachieved in open‐shell planar molecule‐based paramagnets. The structural homogeneity, based on single‐molecular‐weight materials, enabled us to address structural insights and mechanisms via a combination of structural and theoretical investigations.

The designability of d/π‐conjugated molecular complexes,^[^ ^53^, ^54^ ^]^ enabled by tunable ligand and metal combinations^[^ ^33^, ^34^, ^40^ ^]^ and hydrophobic alkyl chains^[^ ^26^, ^33^, ^37^ ^]^ offers significant potential for engineering tailored intermolecular interactions in both aqueous and organic environments. The self‐assembly approach demonstrated in this study establishes a versatile platform for creating stable paramagnetic molecular assemblies with tunable magnetic properties and responsiveness to external stimuli. This paves the way for innovative applications in advanced materials, including nanomedicine and stimuli‐responsive systems. This molecular design strategy expands possibilities for diversifying electronic functionalities in soft conductors, integrating magnetic, electrical, thermal, and optical properties, and represents a significant advancement in the field of soft material sciences.

## Conflict of Interest

The authors declare no conflict of interest.

## Supporting information



Supporting Information

## Data Availability

The data that support the findings of this study are available in the supplementary material of this article.

## References

[1] K.‐R. Naveed , L. Wang , H. Yu , R. S. Ullah , M. Haroon , S. Fahad , J. Li , T. Elshaarani , R. U. Khan , A. Nazir , Polym. Chem. 2018, 9, 3306. 10.1039/c7tb03332j 32254311

[2] S. Kagoshima , H. Nagasawa , T. Sambongi , One‐Dimensional Conductors, Springer Berlin Heidelberg , Berlin, Heidelberg, 1988.

[3] T. Naito , Crystals 2021, 11, 838.

[4] D. Sun , E. Ehrenfreund , Z. Valy Vardeny , Chem. Commun. 2014, 50, 1781. 10.1039/c3cc47126h 24432354

[5] D. Li , G. Yu , Adv. Funct. Mater. 2021, 31, 2100550.

[6] Y. Kim , X. Zhao , Chem. Rev. 2022, 122, 5317. 35104403 10.1021/acs.chemrev.1c00481 PMC9211764

[7] (Eds: M. Kato , K. Ishii ), Soft Crystals: Flexible Response Systems with High Structural Order, Springer, Singapore, 2023. 10.1002/chem.201805641 PMC6593753 30653768

[8] E. Amstad , J. Kohlbrecher , E. Müller , T. Schweizer , M. Textor , E. Reimhult , Nano Lett. 2011, 11, 1664. 21351741 10.1021/nl2001499

[9] M. A. Uddin , H. Yu , L. Wang , B. U. Amin , S. Mehmood , R. Liang , F. Haq , J. Hu , J. Xu , ACS Appl. Mater. Interf. 2021, 13, 61693. 10.1021/acsami.1c21760 34913332

[10] J. Zhou , M. Chen , G. Diao , ACS. Appl. Mater. Interf. 2014, 6, 18538. 10.1021/am5057147 25268246

[11] T. Ren , Q. Liu , H. Lu , H. Liu , X. Zhang , J. Du , J. Mater. Chem. 2012, 22, 12329.

[12] M. Krack , H. Hohenberg , A. Kornowski , P. Lindner , H. Weller , S. Förster , J. Am. Chem. Soc. 2008, 130, 7315. 18484723 10.1021/ja077398k

[13] M. A. Uddin , H. Yu , L. Wang , Y. Sheng , S. Mehmood , B. U. Amin , R. Liang , Mater. Today Commun. 2022, 30, 103107.

[14] E. Yoshida , Colloid. Polym. Sci. 2020, 298, 1205.

[15] A. Kunze , C. T. Murray , C. Godzich , J. Lin , K. Owsley , A. Tay , D. Di Carlo , Lab Chip 2017, 17, 842. 28164203 10.1039/c6lc01349j PMC5400667

[16] C. Sanson , O. Diou , J. Thévenot , E. Ibarboure , A. Soum , A. Brûlet , S. Miraux , E. Thiaudière , S. Tan , A. Brisson , V. Dupuis , O. Sandre , S. Lecommandoux , ACS Nano 2011, 5, 1122. 21218795 10.1021/nn102762f

[17] S. Kim , P. Durand , T. Roques‐Carmes , J. Eastoe , A. Pasc , Langmuir 2015, 31, 1842. 25598433 10.1021/la504708k

[18] S. Kim , C. Bellouard , J. Eastoe , N. Canilho , S. E. Rogers , D. Ihiawakrim , O. Ersen , A. Pasc , J. Am. Chem. Soc. 2016, 138, 2552. 26859700 10.1021/jacs.6b00537

[19] P. Brown , A. Bushmelev , C. P. Butts , J. Cheng , J. Eastoe , I. Grillo , R. K. Heenan , A. M. Schmidt , Angew. Chem., Int. Ed. 2012, 51, 2414. 10.1002/anie.201108010 22266983

[20] P. Brown , C. P. Butts , J. Eastoe , S. Glatzel , I. Grillo , S. H. Hall , S. Rogers , K. Trickett , Soft Matter 2012, 8, 11609.

[21] S. Jeong , J. Park , D. Pathania , C. M. Castro , R. Weissleder , H. Lee , ACS Nano 2016, 10, 1802. 26808216 10.1021/acsnano.5b07584 PMC4802494

[22] T. Kunitake , Angew. Chem. Int. Ed. Engl. 1992, 31, 709.

[23] T. Harayama , H. Riezman , Nat. Rev. Mol. Cell Biol. 2018, 19, 281. 29410529 10.1038/nrm.2017.138

[24] K. Kurihara , M. Tamura , K. Shohda , T. Toyota , K. Suzuki , T. Sugawara , Nat. Chem. 2011, 3, 775. 21941249 10.1038/nchem.1127

[25] E. Sezgin , I. Levental , S. Mayor , C. Eggeling , Nat. Rev. Mol. Cell Biol. 2017, 18, 361. 28356571 10.1038/nrm.2017.16 PMC5500228

[26] M. Hishida , K. Tanaka , Phys. Rev. Lett. 2011, 106, 158102. 21568617 10.1103/PhysRevLett.106.158102

[27] M. Hishida , N. Shimokawa , Y. Okubo , S. Taguchi , Y. Yamamura , K. Saito , Langmuir 2020, 36, 14699. 33232164 10.1021/acs.langmuir.0c02609

[28] D. B. Amabilino , D. K. Smith , J. W. Steed , Chem. Soc. Rev. 2017, 46, 2404. 28443937 10.1039/c7cs00163k

[29] A. J. Savyasachi , O. Kotova , S. Shanmugaraju , S. J. Bradberry , G. M. Ó’Máille , T. Gunnlaugsson , Chem 2017, 3, 764.

[30] A. Jana , S. Bähring , M. Ishida , S. Goeb , D. Canevet , M. Sallé , J. O. Jeppesen , J. L. Sessler , Chem. Soc. Rev. 2018, 47, 5614. 30033473 10.1039/c8cs00035b

[31] H. M. Yamamoto , Y. Kosaka , R. Maeda , J. Yamaura , A. Nakao , T. Nakamura , R. Kato , ACS Nano 2008, 2, 143. 19206558 10.1021/nn700035t

[32] X.‐J. Wang , L.‐B. Xing , W.‐N. Cao , X.‐B. Li , B. Chen , C.‐H. Tung , L.‐Z. Wu , Langmuir 2011, 27, 774. 21142103 10.1021/la103686n

[33] J. W. Grate , S. Rose‐Pehrsson , W. R. Barger , Langmuir 1988, 4, 1293.

[34] M. Ciancone , N. Bellec , S. Cammas‐Marion , A. Dolet , D. Vray , F. Varray , C. L. Goff‐Gaillard , X. L. Goff , Y. Arlot‐Bonnemains , F. Camerel , Langmuir 2019, 35, 15121. 31682444 10.1021/acs.langmuir.9b01296

[35] S. Yokomori , S. Dekura , T. Fujino , M. Kawamura , T. Ozaki , H. Mori , J. Mater. Chem. C 2020, 8, 14939.

[36] M. Ito , T. Fujino , L. Zhang , S. Yokomori , T. Higashino , R. Makiura , K. J. Takeno , T. Ozaki , H. Mori , J. Am. Chem. Soc. 2023, 145, 2127. 36511803 10.1021/jacs.2c08015

[37] G. A. Ferreira , W. Loh , J. Brazil , Chem. Soc. 2016, 27, 392.

[38] Deposition number 2379385 for compound 4 contains the supplementary crystallographic data for this paper. These data are provided free of charge by the joint Cambridge Crystallographic Data Centre and Fachinformationszentrum Karlsruhe Access Structures service via www.ccdc.cam.ac.uk/data_request/cif, or by emailing data_request@ccdc.cam.ac.uk .

[39] A. Guinier , G. Fournet , C. B. Walker , G. H. Vineyard , Phys. Today 1956, 9, 38.

[40] B. Xu , R. Wang , X. Wang , Nanoscale 2012, 4, 2713. 22460235 10.1039/c2nr30139c

[41] S. Yokomori , S. Dekura , A. Ueda , R. Kumai , Y. Murakami , H. Mori , J. Mater. Chem. C 2021, 9, 10718.

[42] H. Ito , Y. Nakahira , N. Ishimatsu , Y. Goto , A. Yamashita , Y. Mizuguchi , C. Moriyoshi , T. Toyao , K. Shimizu , H. Oike , M. Enoki , N. C. Rosero‐Navarro , A. Miura , K. Tadanaga , Bull. Chem. Soc. Jpn. 2023, 96, 1262.

[43] J. Du , R. K. O'Reilly , Soft Matter 2009, 5, 3544.

[44] Q. Yu , J.‐Y. Ge , Z.‐P. Lv , H.‐Y. Wang , J.‐L. Zuo , RSC. Adv. 2016, 6, 100783.

[45] D. W. Breiby , S. Sato , E. J. Samuelsen , K. Mizoguchi , J. Polym. Sci. B Polym. Phys. 2003, 41, 3011.

[46] N. Kinoshita , M. Tokumoto , H. Anzai , G. Saito , J. Phys. Soc. Jpn. 1985, 54, 4498.

[47] M. Fujiwara , T. Kambe , K. Oshima , Synthet. Met. 2005, 153, 489.

[48] D. Vušak , K. Mišković Špoljarić , J. Jurec , D. Žilić , B. Prugovečki , Croat. Chem. Acta 2023, 95, 157.

[49] Y. Misaki , T. Kaibuki , M. Taniguchi , K. Tanaka , T. Kawamoto , T. Mori , T. Nakamura , Chem. Lett. 2000, 29, 1274.

[50] K. Yamazoe , Y. Higaki , Y. Inutsuka , J. Miyawaki , Y.‐T. Cui , A. Takahara , Y. Harada , Langmuir 2017, 33, 3954. 28359152 10.1021/acs.langmuir.7b00243

[51] S. Yamamoto , Y. Senba , T. Tanaka , H. Ohashi , T. Hirono , H. Kimura , M. Fujisawa , J. Miyawaki , A. Harasawa , T. Seike , S. Takahashi , N. Nariyama , T. Matsushita , M. Takeuchi , T. Ohata , Y. Furukawa , K. Takeshita , S. Goto , Y. Harada , S. Shin , H. Kitamura , A. Kakizaki , M. Oshima , I. Matsuda , J. Synchrotron. Rad. 2014, 21, 352. 10.1107/S1600577513034796 PMC3945419 24562556

[52] H. Wang , C. Y. Ralston , D. S. Patil , R. M. Jones , W. Gu , M. Verhagen , M. Adams , P. Ge , C. Riordan , C. A. Marganian , P. Mascharak , J. Kovacs , C. G. Miller , T. J. Collins , S. Brooker , P. D. Croucher , K. Wang , E. I. Stiefel , S. P. Cramer , J. Am. Chem. Soc. 2000, 122, 10544.

[53] G. C. Papavassiliou , G. C. Anyfantis , G. A. Mousdis , Crystals 2012, 2, 762.

[54] R. Kato , Chem. Rev. 2004, 104, 5319. 15535652 10.1021/cr030655t

