# Supplementary material for: Macroscopic Structural Transition of Nickel Dithiolate Capsule with Uniaxial Magnetic Anisotropy in Water
Source: Adv Sci (Weinh). 2025 Apr 23;12(29):2504967. doi: 10.1002/advs.202504967 (PMC12362769; doi:10.1002/advs.202504967)
Supplement: Supplementary file 1 — Supporting Information [file ADVS-12-2504967-s001.pdf]

ADVANCED SCIENCE
Open Access

Supporting Information

for *Adv. Sci.*, DOI 10.1002/advs.202504967

Macroscopic Structural Transition of Nickel Dithiolate Capsule with Uniaxial Magnetic Anisotropy in Water

Tomoko Fujino, Mafumi Hishida*, Masatoshi Ito, Toshikazu Nakamura, Mizue Asada, Naoya Kurahashi, Hisao Kiuchi, Yoshihisa Harada, Koji Harano, Rie Makiura, Kanokwan Jumtee Takeno, So Yokomori, Hiroshi Oike and Hatsumi Mori*

Supporting Information

Macroscopic Structural Transition in a Nickel Dithiolate Capsule with Uniaxial Magnetic Anisotropy in Water

Tomoko Fujino[a]*, Mafumi Hishida[b]*, Masatoshi Ito[a], Toshikazu Nakamura[c], Mizue Asada[c], Naoya Kurahashi[a], Hisao Kiuchi[a,d], Yoshihisa Harada[a,d], Koji Harano[e,f], Rie Makiura[g], Kanokwan Jumtee Takeno[g], So Yokomori[a], Hiroshi Oike[h], and Hatsumi Mori[a]

[a]*The Institute for Solid State Physics, The University of Tokyo, 5-1-5 Kashiwanoha, Kashiwa, Chiba 277-8581, Japan*
[b]*Department of Chemistry, Faculty of Science, Tokyo University of Science, 1-3 Kagurazaka, Shinjuku, Tokyo 162-8601, Japan*
[c]*Institute for Molecular Science, 38 Nishigo-Naka, Myodaiji, Okazaki, Aichi 444-8585, Japan*
[d]*Synchrotron Radiation Collaborative Research Organization, The University of Tokyo, Sendai, Miyagi 980-8572, Japan*
[e]*Center for Basic Research on Materials, National Institute for Materials Science (NIMS), 1-1 Namiki, Tsukuba, Ibaraki 305-0044, Japan*
[f]*Research Center for Autonomous Systems Materialogy (ASMat), Institute of Integrated Research, Institute of Science Tokyo, 4259 Nagatsuda-cho, Midori-ku, Yokohama, Kanagawa 226-8501, Japan*
[g]*Department of Materials Science, Osaka Metropolitan University, Gakuen-cho, Naka-ku, Sakai, Osaka 599-8570, Japan*
[h]*PRESTO, Japan Science and Technology Agency (JST), Kawaguchi, Saitama 332-0012, Japan*

***Corresponding author**
Email: fujino@issp.u-tokyo.ac.jp (Tomoko Fujino) and hishida@rs.tus.ac.jp (Mafumi Hishida)

Table of Contents

1. General S2
2. Methods S2
3. Materials S2
4. Synthesis S2
    4-1. Synthesis of Ph_4P·**Ni(2OMe)** S2
    4-2. Synthesis of DDA·**Ni(2OMe)** S3
    4-3. Assembly of DDA·**Ni(2OMe)** in water S3
5. Theoretical calculations for Ni(2OMe) anion in the isolated state S3
6. Single-crystal XRD analysis S4
7. SAXS and WAXS analyses S5
8. TEM observation S9
9. DLS analyses S12
10. ESR measurements S12
11. UV analysis S14
12. XAS analysis S17
13. Coordinates of optimized structures S19
14. Supplementary references S26

1. General

All variations of oxygen- and moisture-sensitive materials were synthesized under an argon atmosphere.

2. Methods

Mass spectra (MS) were obtained using a JEOL JMS-AX500 with a field desorption (FD) probe in positive mode. Single-crystal X-ray diffraction (XRD) measurements were performed using a Rigaku MercuryII CCD X-ray diffractometer (Mo K_α, λ = 0.71073 Å). Elemental analysis for the CHN compositions was performed using an Elementar Vario MICRO CUBE analyzer. The S composition was analyzed using a Thermo Fisher Scientific ICS-1600 analyzer. Small- and wide-angle X-ray scattering (SAXS and WAXS) measurements were performed simultaneously at BL10C in the Photon Factory at the High Energy Accelerator Research Organization (KEK), Japan, with detectors PILATUS3 2M for SAXS and PILATUS3 200K for WAXS (DECTRIS Ltd., Baden, Switzerland). Transmission electron microscopy (TEM) observation was carried out on a Thermo Fisher Scientific Talos F200X G2 operated at an accelerating voltage of 80 kV under 5×10^{-6} Pa in a specimen column. TEM images were recorded under an underfocus condition (defocus value: 1–2 μm) with an exposure time of 1.0 s on a Ceta-D camera. The X-band (approximately 9.4 GHz) continuous wave electronic spin resonance (ESR) experiments were performed using Bruker EMXmicro (powder and dichloromethane solutions) and EMX (aqueous solutions). Dynamic light scattering (DLS) measurement was conducted on a Malvern Zetasizer Nano ZS machine. UV-Vis-NIR spectra were recorded on the JASCO V-670 spectrophotometer with a Peltier temperature controller. Ni L-edge X-ray absorption spectroscopy (XAS)[S1] was performed at the beamline BL07LSU HORNET[S2] end station in SPring-8 synchrotron facilities.

3. Materials

The following reagents were purchased from commercial suppliers and used as received: sodium methoxide (Kanto Chemical), nickel(II) dichloride hexahydrate (Kanto Chemical), nickel(II) diacetate tetrahydrate (Wako Pure Chemical Industries), acetone (superhydrated, Wako Pure Chemical Industries), methanol (superdehydrated, Wako Pure Chemical Industries), didodecyldimethylammonium (DDA) chloride (Tokyo Chemical Industry). Water was purified by Milli-Q ultrapure water systems (Merck Direct-Q UV5 and EQ7000).

4. Synthesis

4-1. Synthesis of Ph_4P·Ni(2OMe) 4

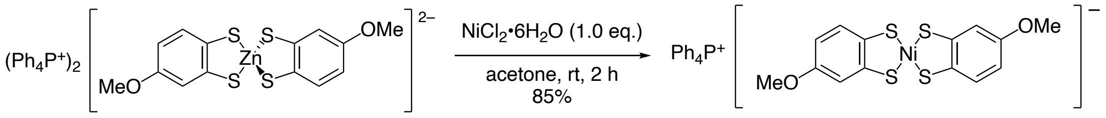

3 4

To a suspended solution of Zn(II) complex **3**[S3] (273 mg, 0.252 mmol) in acetone 50 mL was added nickel(II) dichloride hexahydrate (60.3 mg, 0.254 mmol), and the mixture was stirred at room temperature for 2 h. After the precipitate had been removed by celite filtration, the solution was concentrated in vacuo. The material was mixed with ethanol (10 mL) and subjected to ultrasonic irradiation. The precipitate was collected by filtration to afford green powder **4** (157 mg, 0.213 mmol, 85%). Green single crystals were obtained by solvent evaporation with acetone/ethanol (10:1, v/v). The structural integrity was confirmed by the single-crystal XRD structure analysis (Table S1, Figures 3c, and S1), confirming the valency of the

Ni(2OMe) anion to be −1. It is noted that direct complexation between **2** and divalent nickel species, such as nickel(II) diacetate tetrahydrate, similar to the synthesis of **1** described below, was achievable; however, the reaction proved to be unreproducible, likely due to the instability of the intermediate compound during the reaction process.

4-2. Synthesis of DDA·Ni(2OMe) 1

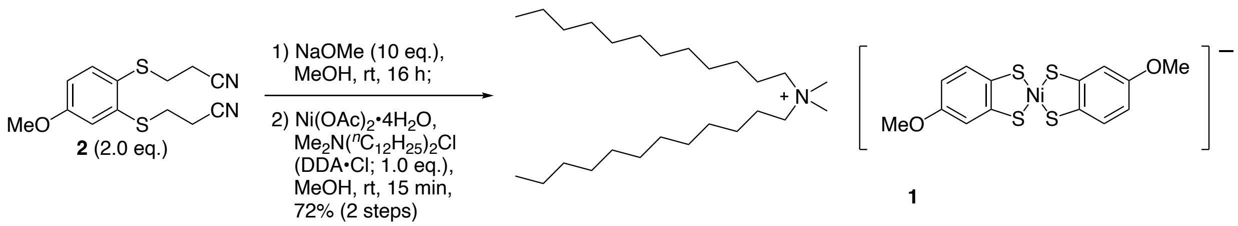

To a suspended solution of thioether **2** (200 mg, 0.719 mmol) in methanol (15 mL) was added sodium methoxide (194 mg, 3.59 mmol), and the mixture was stirred at ambient temperature for 16 h. After the addition of nickel(II) diacetate tetrahydrate (90.5 mg, 0.364 mmol) and DDA chloride (152 mg, 0.363 mmol), the mixture was stirred at room temperature for 15 min. The reaction was quenched with Milli-Q water (50 mL). The precipitate was collected by filtration, rinsing with Milli-Q water and methanol, and drying in vacuo to afford a green powder **1** (204 mg, 0.261 mmol, 72% for two-step transformation from **2**). The structural integrity of **1** was determined by mass spectrometry with a FD probe in positive mode; MS calcd for $C_{40}H_{68}NNiO_2S_4^+$ [**M**]$^{\bullet+}$ 780.35, found 780.34 and $C_{66}H_{124}N_2NiO_2S_4$ [**M** + DDA]$^+$ 1162.79, found 1162.78. The composition was supported by the elemental analysis (CHNS composition) using calibration curves based on the integrated intensity obtained: calcd for DDA·**Ni(2OMe) 1** ($C_{40}H_{68}NNiO_2S_4$) C 61.44%; H 8.77%; N 1.79%; S 16.40% and $(DDA)_2$·**Ni(2OMe)** ($C_{40}H_{68}NNiO_2S_4$) C 68.06%; H 10.73%; N 2.41%; S 11.01%, found: C 61.26%; H 7.78%; N 2.62%; S 13.61%. The data indicates that the dominant composition product was confirmed to be DDA·**Ni(2OMe) 1**, implying the primary valency of the **Ni(2OMe)** complex is −1 (open-shell), as apparent in ESR spectra (Figures 7 and S8).

4-3. Assembly of DDA·Ni(2OMe) 1 in water

In a 100 mL vial, a solution of **1** (22.7 mg, 29.1 µmol) in dichloromethane (3.0 mL) was evaporated while stirring in vacuo. To the film in the vial, Milli-Q water (29 mL) was added, and the vial was heated while stirring on a 120 °C hot plate for 10 min (the sample temperature was measured to be 90 °C). The mixture was subjected to ultrasonic irradiation (40 kHz, AS ONE MUC-63) for 120 sec. After heating the sample on a 120 °C hot plate, the mixture was subjected to ultrasonic irradiation from a homogenizer (20 kHz, SMT UH-50) for 4 × 120 sec. The sample was allowed to cool to ambient temperature over 12 h. The sample was allowed to cool to ambient temperature over 12 h. After removal of the yellowish-green precipitate by collecting supernatant, we obtained the whitish-green suspension (25 mL; approximately 1 µM).

5. Theoretical calculations for Ni(2OMe) anion in the isolated state

The singly occupied molecular orbital (SOMO) of the **Ni(2OMe)** anion was calculated based on density functional theory (DFT) at the B3LYP/6−311G++ (d,p) level (SDD for Ni) using the Gaussian16 program,[S4] based on the optimized atomic coordinates (Table S3). The frontier orbitals are depicted in Figure 3b.

6. Single-crystal XRD analysis

The single-crystal XRD analysis of Ph_4P·**Ni(2OMe) 4** (Table S1 and Figure S1a) was performed. The structures were analyzed using a direct method (SHELXT[S5] version 2018/2) and refined with a full-matrix least-squares technique (SHELXL version 2018/3) using an Olex2[S6]-1.2 (OlexSys) software. Anisotropic thermal parameters were applied to all non-hydrogen atoms, and the hydrogen atoms were generated geometrically. The unit cell consists of two crystallographically independent half-molecule anions and two cations. The bond lengths of the two anions are shown in Figures 3c and S1b.

Table S1. Crystallographic data for single-crystal Ph_4P·**Ni(2OMe) 4**.

Compound	Ph_4P·**Ni(2OMe) 4**
Temperature / K	293
Formula	$C_{38}H_{32}NiO_2PS_4$
Formula weight	738.55
Crystal system	*triclinic*
Space group	P–1 (#2)
a / Å	10.077(3)
b / Å	11.796(3)
c / Å	15.601(4)
α / deg.	105.031(3)
β / deg.	94.374(3)
γ / deg.	100.261(3)
V / Å^3	1747.8(8)
Z	2
D_{calc} / g cm^{-3}	1.403
R_{int}	0.0243
R_1 ($I > 2.00\sigma(I)$)	0.0505
wR_2 (all reflections)	0.1371
GOF	1.059
CCDC	2379385

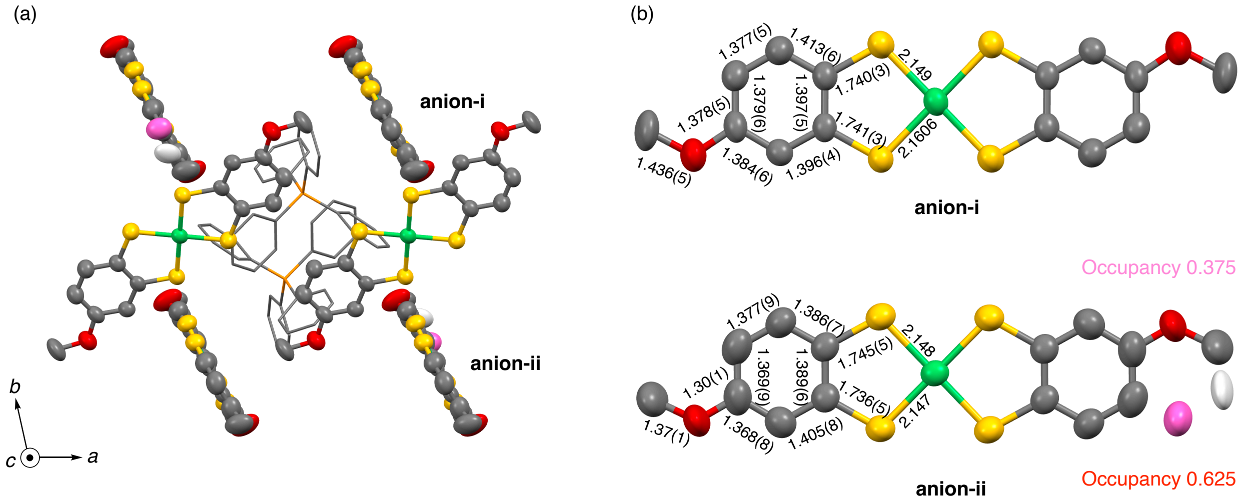

Figure S1. Single-crystal structure of Ph_4P·**Ni(2OMe) 4**. (a) The packing structures along the *c*-axis. (b) The anion structures with bond lengths (Å) are displayed perpendicular to the π-plane of the molecule. The anion structures are presented in ORTEP (50% thermal ellipsoid), and Ph_4P cations are displayed in capped sticks. Hydrogens are omitted for clarity. Atoms are colored as follows: nickel: green; oxygen: red; sulfur: yellow; carbon: gray; phosphine: orange. Disordered oxygen and carbon atoms in minor occupancy are colored pink and light gray, respectively.

7. SAXS and WAXS analyses

Temperature-dependent SAXS and WAXS of suspension **1a** (approximately 1 μM) were measured simultaneously with an incident X-ray with a wavelength of 1.5 Å. The temperature-dependent measurements were performed using a temperature controller (FP900, Mettler-Toledo Inc., Columbus, OH). The measured q range of SAXS was 0.006 $Å^{-1}$ to 0.36 $Å^{-1}$ at a sample-to-detector distance of 1.1 m. The range of WAXS was 0.8 $Å^{-1}$ to 2.0 $Å^{-1}$ at a sample-to-detector distance of 0.26 m. The sample-to-detector lengths and tilt angles of the detectors were precisely calibrated using the data of a standard sample (silver behenate). After calibration, circular averages for the obtained 2D images were performed using Nika[S7] to generate one-dimensional diffraction profiles. Background (SAXS profile of H_2O) was reduced (Figures 4 and S4) on the one-dimensional diffraction profiles. The temperature-dependent intensity change is shown in Figure S3. The profiles of suspension **1a** and **1c** at 10 °C was initially analyzed by a three-layer model using SasView software,[S8] which did not fit well. Therefore, we ultimately adopted a five-layer core-shell model. Here, we applied five shells for the bilayer form factor, considering two nickel dithiolate ions, two ammonium groups and a hydrophobic layer. This model is also physically reasonable because it appropriately separates the hydrophilic headgroups and anions of the surfactant. As shown in Figure S2, SAXS profiles likely reflect a form factor of a bilayer membrane. The solid lines in Figure S2a,b are the calculated results, and Figure S2c,d represent the corresponding electron densities of **1a** and **1c**. Figure S2e,f are the schematic images of assemblies **1a** and **1c**. The scattering peak around 0.25 $Å^{-1}$ is considered to be derived from precipitated or aggregated components (lamellar crystalline phase). In addition, the lack of clear oscillations in the scattering profile is likely due to the polydispersity of the membrane structure. The time-dependent structural transition in the SAXS profile at ambient temperature was analyzed following brief heating at 90 °C for 5 min and subsequent cooling to ambient temperature (Figure S4).

WAXS measurements (Figure S5) showed a scattering peak at q of approximately 1.5 $Å^{-1}$ (corresponding to 4.2 Å) and 1.6 $Å^{-1}$ (corresponding to 3.9 Å) in **1a** at low temperatures, as depicted with arrows. The former and the latter likely correspond to the in-plane alkyl chain ordering of DDA and the stacking of the **Ni(2OMe)** anions. Specifically, the peak at $q = 1.6$ $Å^{-1}$ corresponds to $d \approx 3.9$ Å, a reasonable value for the distance between stacked aromatic anions. This suggests that the ordering of anions influences the magnetic

properties and is a primary structural factor for the electronic functionality of this material with unique uniaxial anisotropic magnetism (Figures 7 and S8). While we cannot completely rule out the possibility that these peaks originate from different repeating distances in the bilayer or other structural features, the potential for a highly ordered stacking structure of anions is most likely, considering the unique magnetism observed in this study. In **1c**, a significant peak at q of 1.5 Å^{-1} was exclusively observed at low temperatures, suggesting that the elongation of anion–anion distances in the interdigitated membrane may weaken the anion stacking interaction (Figures 5 and S2f).

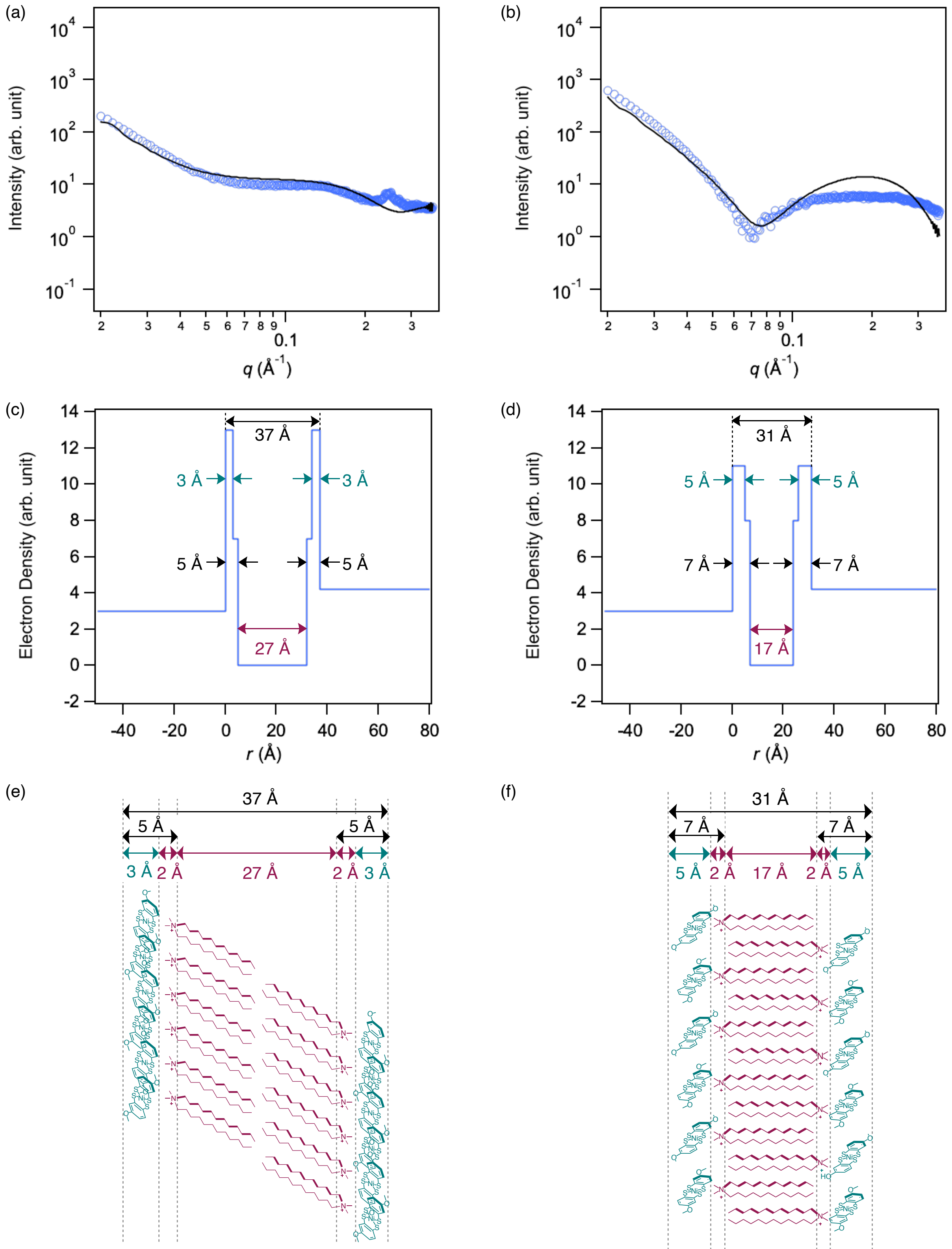

Figure S2. The radial electron density (ED) distribution profile (a,b) and simulated thickness (c,d), corresponding to the possible orientations (c,d), for **1a** (a,c,e) and **1c** (b,d,f).

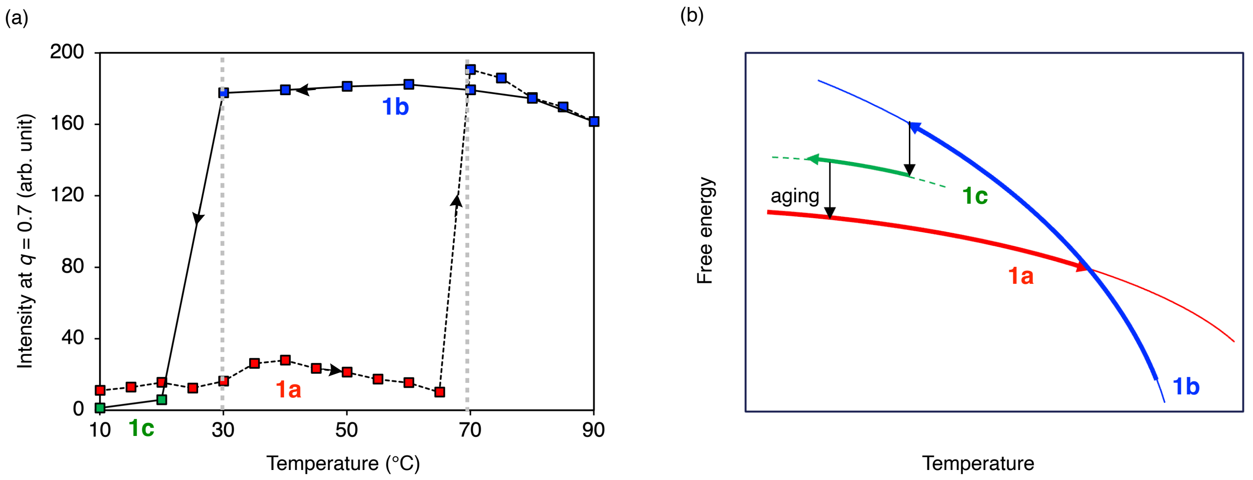

Figure S3. (a) Temperature-dependent intensity change in the SAXS data (Figure 3) at $q = 0.7$. (b) Possible energy diagram for temperature dependency.

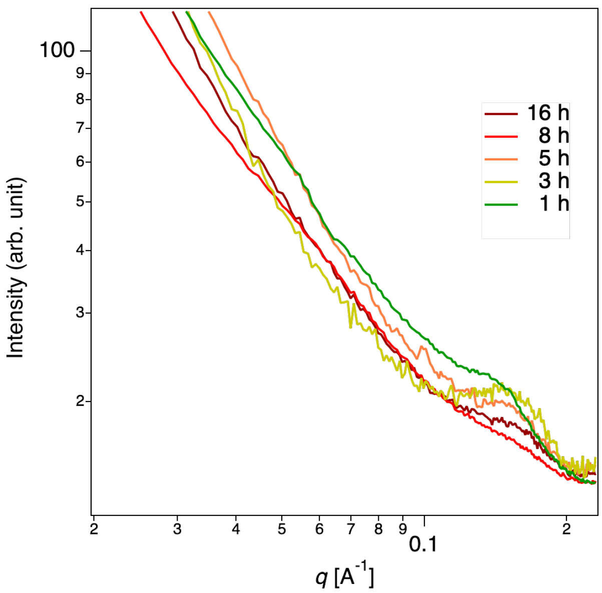

Figure S4. Time-dependent SAXS data from **1c** to **1a**. After 3 h, assembly **1a** was recovered.

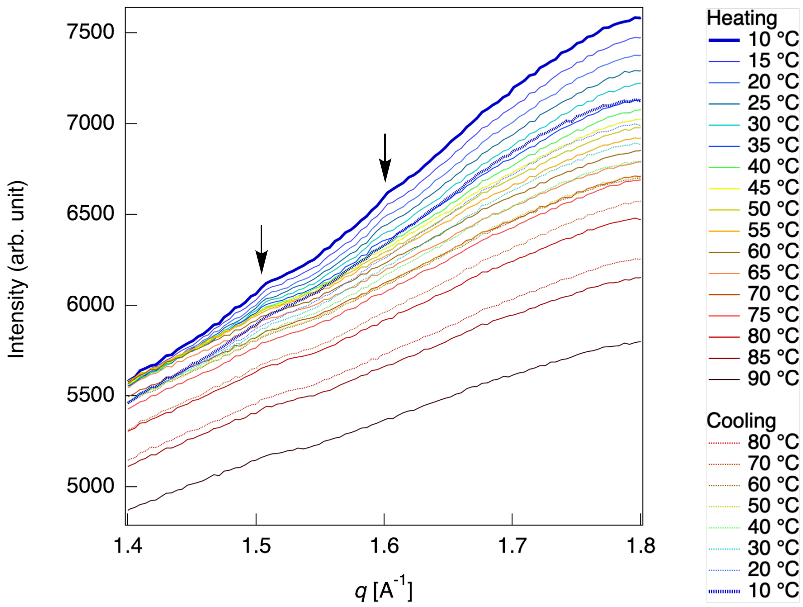

Figure S5. Temperature-dependent WAXS data showing the changes in forms from **1a** through **1b** to **1c**.

8. TEM observation

TEM observations of **1a** and **1c** were prepared as follows. The approximately 1 μM suspensions of **1a** and **1c** were diluted 100-fold (i.e., approximately 10 nM) with Milli-Q water. The supernatant was removed, and the bottom layer (< 0.05 mL) was mess-up to 1 mL with Milli-Q water. A portion of the resulting suspension (2 μL) was placed on a TEM microgrid covered with an amorphous carbon film (SHR-C075, Okenshoji Co., Ltd.) and dried thoroughly under an atmospheric pressure and then under vacuum (66 Pa) for 30 min. TEM observations were carried out on a Thermo Fisher Scientific Talos F200X G2 operated at an accelerating voltage of 80 kV under 5×10^{-6} Pa in the specimen column. TEM images were recorded under an underfocus condition (defocus value: 1–2 μm) with an exposure time of 1.0 sec on the Ceta-D camera (Figures 6 and S6). All images were processed using Velox software (Thermo Fisher Scientific).

(a)

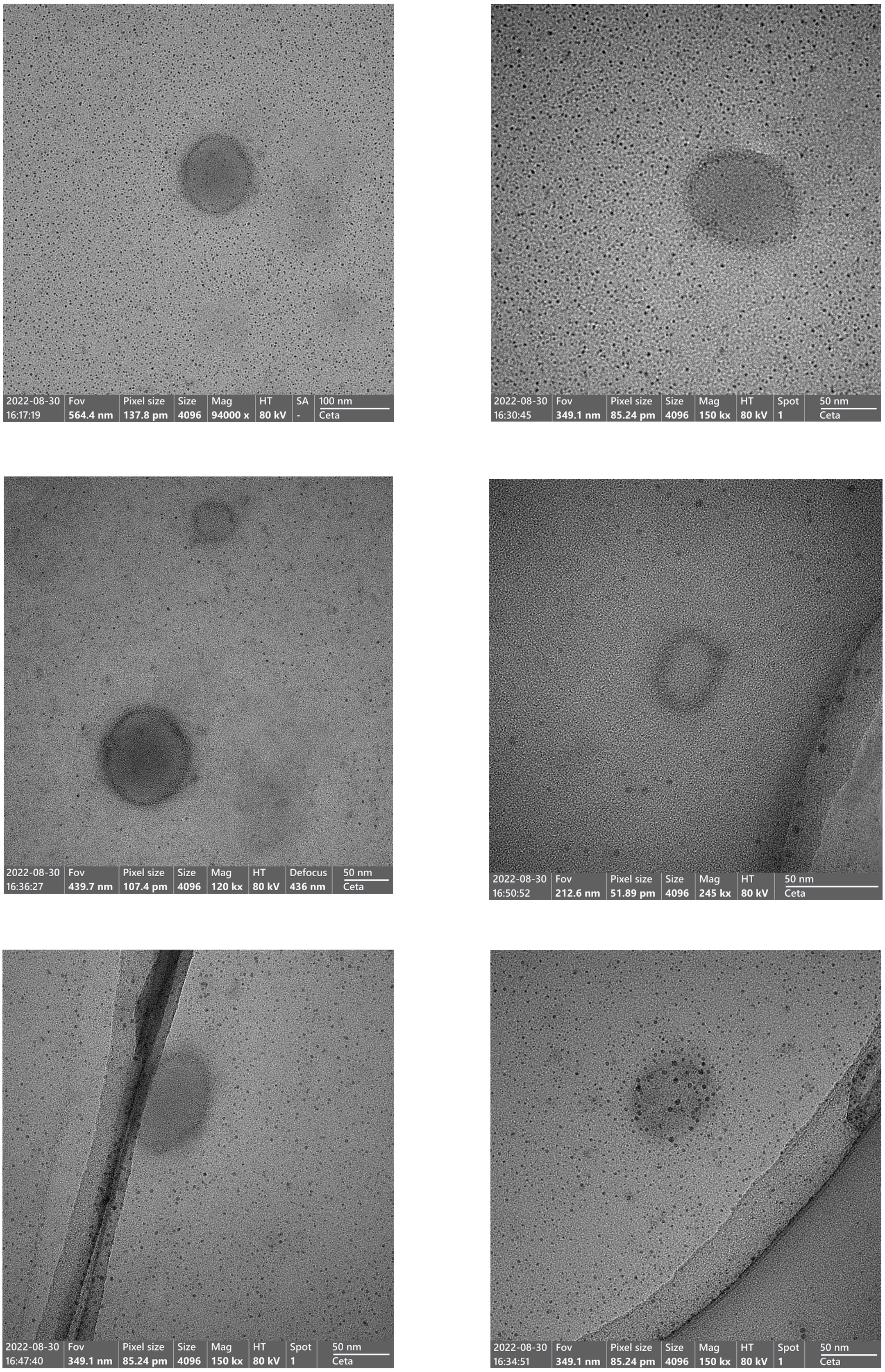
2022-08-30 16:17:19 | Fov 564.4 nm | Pixel size 137.8 pm | Size 4096 | Mag 94000 x | HT 80 kV | SA - | 100 nm | Ceta
2022-08-30 16:30:45 | Fov 349.1 nm | Pixel size 85.24 pm | Size 4096 | Mag 150 kx | HT 80 kV | Spot 1 | 50 nm | Ceta
2022-08-30 16:36:27 | Fov 439.7 nm | Pixel size 107.4 pm | Size 4096 | Mag 120 kx | HT 80 kV | Defocus 436 nm | 50 nm | Ceta
2022-08-30 16:50:52 | Fov 212.6 nm | Pixel size 51.89 pm | Size 4096 | Mag 245 kx | HT 80 kV | 50 nm | Ceta
2022-08-30 16:47:40 | Fov 349.1 nm | Pixel size 85.24 pm | Size 4096 | Mag 150 kx | HT 80 kV | Spot 1 | 50 nm | Ceta
2022-08-30 16:34:51 | Fov 349.1 nm | Pixel size 85.24 pm | Size 4096 | Mag 150 kx | HT 80 kV | Spot 1 | 50 nm | Ceta

(b)

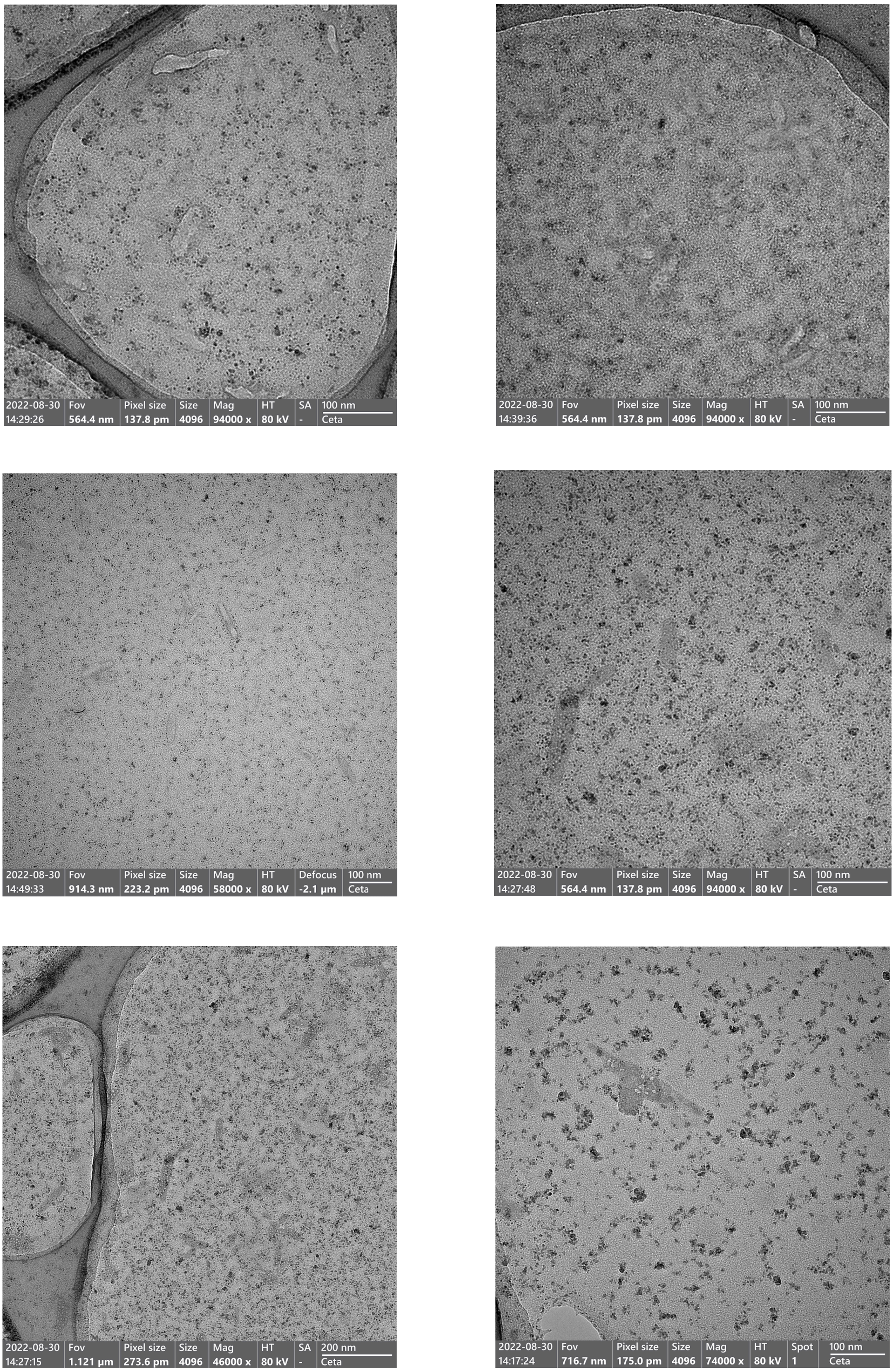

Figure S6. TEM images of dried samples of **1a** (a) and **1c** (b) on films supported by a TEM microgrid.

9. DLS analyses

DLS measurements were performed on a Malvern Zetasizer Nano ZS equipped with a He-Ne laser operating at 4 mW power and 633 nm wavelength and a computer-controlled correlator at a 173° accumulation angle. Temperature-dependent DLS analyses of suspension **1a** (approximately 1 µM) were performed in a plastic cuvette. The measurements were conducted while heating **1a** from 15 to 85 °C to dissociate it to **1** and then cooling to 15 °C to form **1c**. Samples were equilibrated for 2 min at the set temperature. The data were processed using Dispersion Technology software (ver. 5.10) to provide the particle size distribution and average particle sizes through CONTIN analysis. The data were obtained as an average of duplicate or triplicate experiments (Figure S7). The average sizes of **1a** and **1c** were estimated to be approximately 55 nm. The temperature dependency of **1a** indicates that the aggregates dispersed into relatively small-sized particles (approximately 25 nm) at the high temperature.

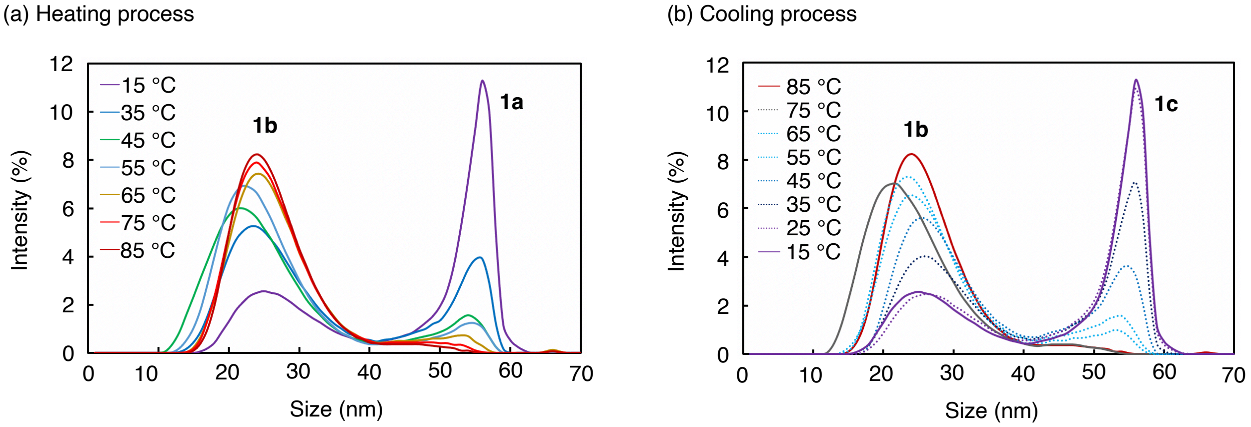

Figure S7. Temperature-dependent DLS measurements of assembly of **1**.

10. ESR measurements

The X-band (~9.5 GHz) continuous wave ESR experiments were conducted on assemblies **1a** and **1c** (Figures 7a and S8b,c). Milli-Q water and powder (polycrystalline) of **1** were used as references (Figure S8a,d). The ESR spectra for suspension (approximately 1 µM) of **1a** and **1c** were similar, highlighting the resemblance in their electronic spin structures. The spectra for **1a**/**1c** and polycrystalline **1** were simulated using EasySpin software[S9] (ver. 6.0.4) based on the measured microwave (MW) frequency (Figure S8e,f). The simulations revealed two distinct *g* values (2.03 and 2.17) for **1a**/**1c**, indicating uniaxial anisotropic magnetism, and three *g* values (2.01, 2.05, and 2.15) for polycrystalline **1**, indicating typical triaxial anisotropic magnetism.

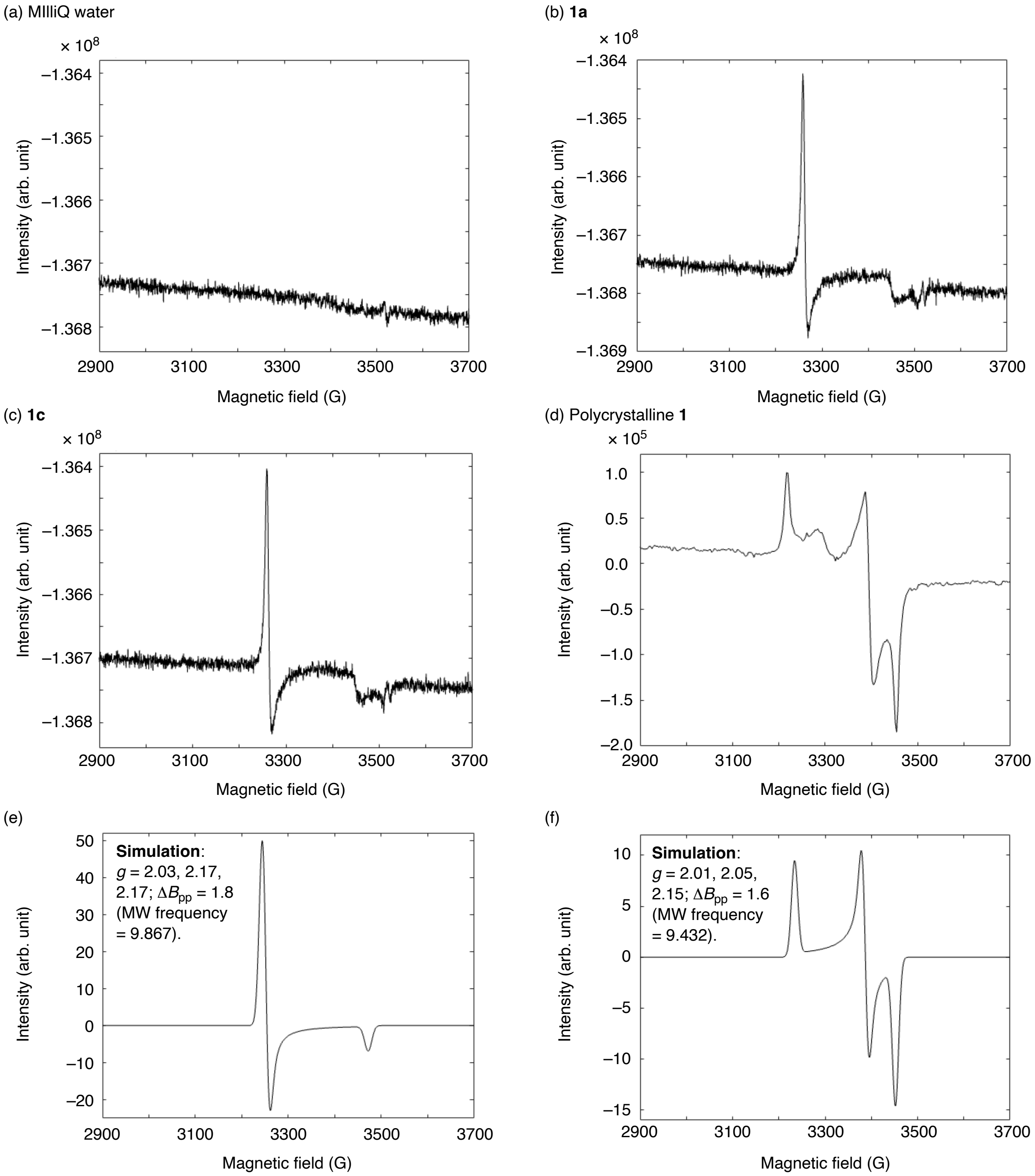

Figure S8. ESR spectra of Milli-Q water (a), **1a** (b), **1c** (c), polycrystals of **1**, (d) and simulated spectra for **1a**/**1c** (e) and polycrystals (f) using setting g and ΔB_{pp} values at the experimentally observed microwave (MW) frequency.

11. UV analysis

Temperature-dependent absorption spectra of suspension **1a** (approximately 1 μM) in water were measured in a quartz cell. The measurements were conducted while heating the sample from 20 to 80 °C (i.e., assembly **1a** to the dissociated **1b**; Figure S9a) and subsequently cooling from 80 °C to 20 °C (i.e., the dissociated **1b** to assembly **1c**; Figure S9b). The UV spectra of **1a** and **1c** were similar, highlighting the resemblance in their electronic spin structures, regardless of the differing macroscopic structures, as apparent in the ESR spectra (Figures 7 and S8) and Ni L-edge X-ray XAS (Figure 8). During the heating of **1a** to **1b** in water at high temperatures, a decrease in the absorbance at 900 nm and the appearance of the absorbance at approximately 960 nm were observed. The appearance may indicate that the charge transfer occurs between the metal and ligands, potentially driven by hydrogen bond formations with surrounding water molecules.[S3]

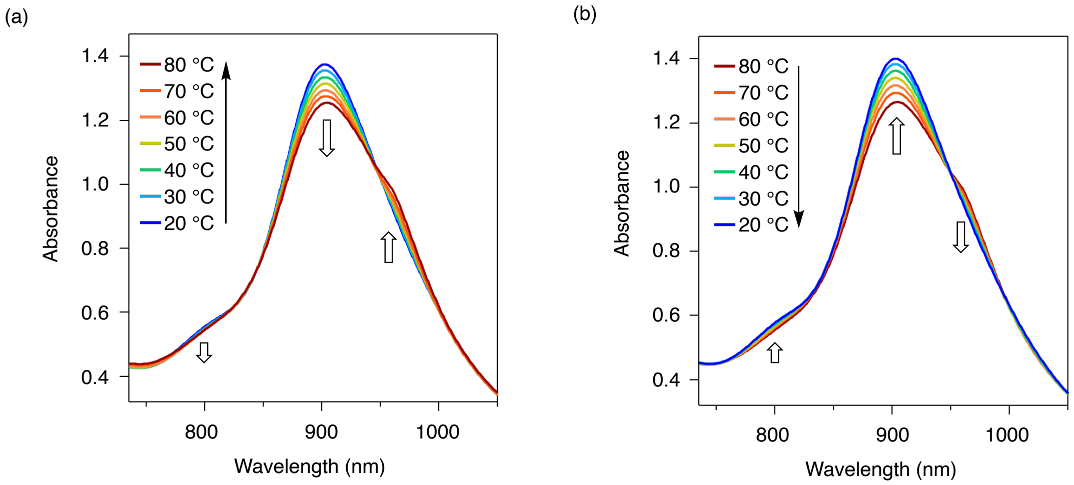

Figure S9. Temperature-dependent UV spectra of **1a** during heating (a) and cooling (b).

We simulated the absorption spectra for the water molecule adducts to the anion to investigate the possible mechanism involving the hydrogen bond formation between the **Ni(2OMe)** anion and water molecules. Two types of water adducts (i.e., **Ni(2OMe)**$^-$·H_2O (i) and **Ni(2OMe)**$^-$·H_2O (ii); Figure S10a,c, Tables S4, and S6) were structure-optimized using DFT at the UB3LYP/6−311G++ (d,p) (SDD for Ni) level with the Gaussian16 program.[S4] Notably, when alternative structural candidates were considered, including a water molecule adduct forming a hydrogen bond with either a sulfur atom in the one-side ligand or the nickel atom, the DFT-optimization consistently converged to **Ni(2OMe)**$^-$·H_2O (i).

Subsequently, the absorption spectra for water adducts **Ni(2OMe)**$^-$·H_2O (i), **Ni(2OMe)**$^-$·H_2O (ii), as well as the intrinsic **Ni(2OMe)**$^-$ (Table S3), were simulated using TDDFT calculations (Figure S10a). The comparison of the simulated spectra revealed that **Ni(2OMe)**$^-$·H_2O (i) predicts a bathochromic shift relative to the intrinsic **Ni(2OMe)**$^-$, whereas **Ni(2OMe)**$^-$·H_2O (ii) predicts a comparable or hypsochromic shift (Figure S10a). Based on these simulations, the presence of a peak at the higher wavelength in the observed UV spectra at elevated temperatures (Figure S9) supports the formation of **Ni(2OMe)**$^-$·H_2O (i).

To further elucidate the origin of the spectral changes—whether arising from geometric or electronic contributions due to water molecule addition—we simulated the spectra of water-depleted structures derived from the water-adducts (i.e., **Ni(2OMe)**$^-$·H_2O (i) – H_2O and **Ni(2OMe)**$^-$·H_2O (ii) – H_2O; Figure S10b,d,

Tables S5, and S7). The assignment of the transitions for the spectra were listed in Table S2. The bathochromic or hypochromic shifts were observed only in the simulated spectra for the water adducts, and water-depleted structures did not exhibit significant shifts (Figure S11b,c). These results indicate that the spectral shifts are primarily due to electronic contributions, specifically charge transfers associated with hydrogen bond formation, rather than geometric factors. This conclusion is further supported by the consistent spectra of **Ni (2OMe)**$^{-}$·H_2O (i) – H_2O, **Ni(2OMe)**$^{-}$·H_2O (ii) – H_2O, and **Ni(2OMe)**$^{-}$ (Figure S11d).

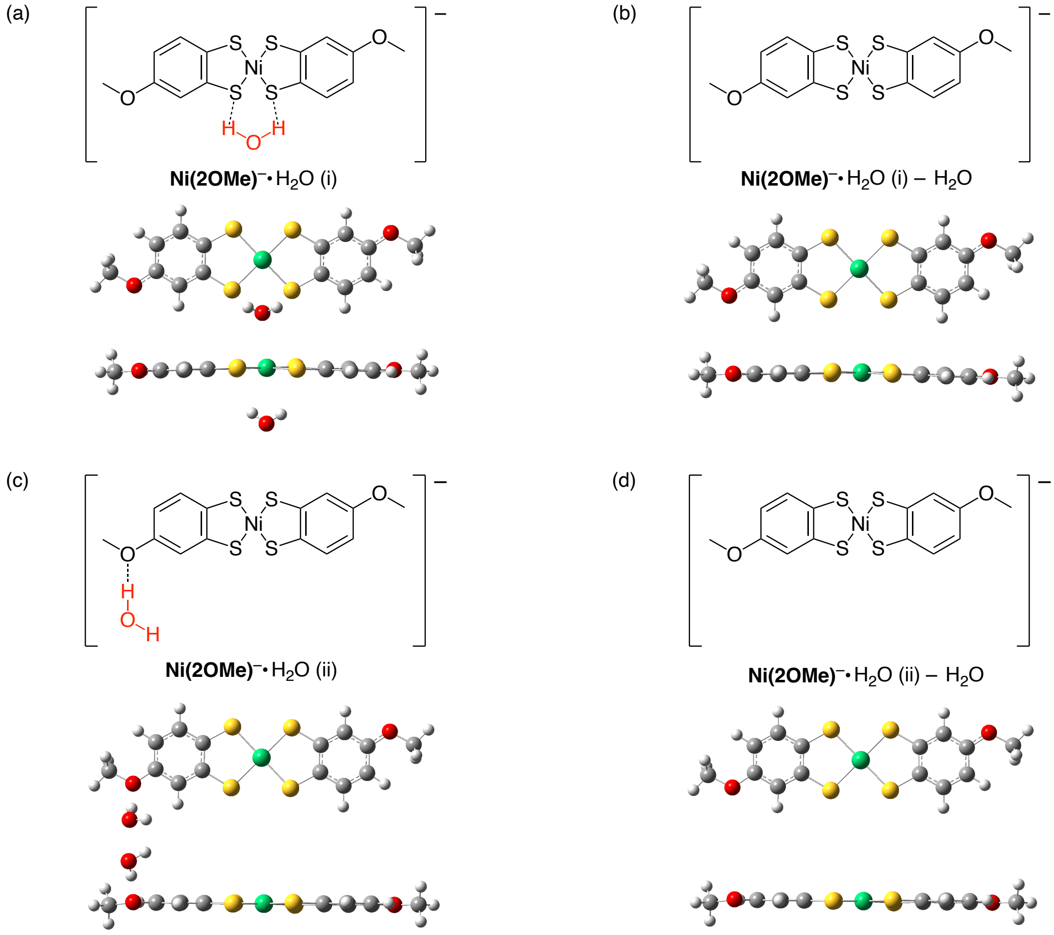

Figure S10. DFT-optimized structure of water molecule-adducts to **Ni(2OMe)** anion (a,c) and the water molecule-depleted structure from the adducts (b,d). Molecular structures (top), the top view of DFT-optimized structures (middle), and the side view of DFT-optimized structures (bottom). Atoms are colored as follows: nickel: green, carbon: gray, sulfur: yellow, oxygen: red, hydrogen: white.

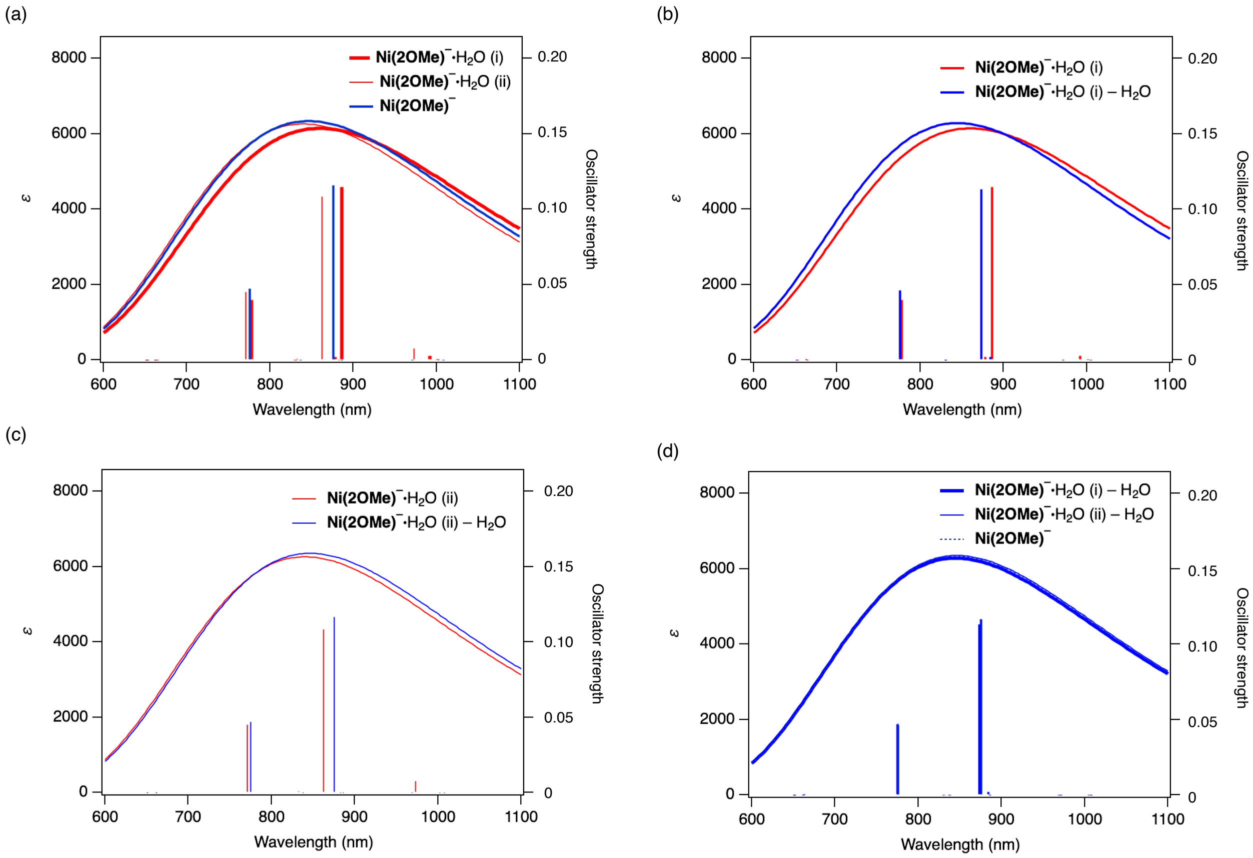

Figure S11. TDDFT-calculated molecular extinction coefficient (ε; lines) and oscillator strength (bars) for the **Ni(2OMe)** anion with (red) and without (blue) water molecules. (a) **Ni(2OMe)**$^-$·H_2O (i), **Ni(2OMe)**$^-$·H_2O (ii), and intrinsic **Ni(2OMe)**$^-$. (b) **Ni(2OMe)**$^-$·H_2O (i) and its water-depleted counterpart **Ni(2OMe)**$^-$·H_2O (i)–H_2O. (c) **Ni(2OMe)**$^-$·H_2O (ii) and its water-depleted counterpart **Ni(2OMe)**$^-$·H_2O (ii)–H_2O. (d) Water-depleted **Ni(2OMe)**$^-$·H_2O (i)–H_2O, **Ni(2OMe)**$^-$·H_2O (ii)–H_2O, along with intrinsic **Ni(2OMe)**$^-$.

Table S2. The absorption wave lengths (λ) and oscillator strengths (f) with transition configurations of for the **Ni(2OMe)** anion with and without water molecules calculated by TDDFT method (UB3LYP/6−311G++ (d,p) (SDD for Ni) level).

Compound	λ (nm)	f	Transition configuration (CI coefficient)
**Ni(2OMe)**$^{-}$	876.23	0.1158	94A (HOMO–3-α) → 99A (LUMO-α) (0.12318)<br>97B (HOMO-β) → 98B (LUMO-β) (0.97736)
	775.94	0.0473	94A (HOMO–3-α) → 99A (LUMO-α) (0.10029)<br>95B (HOMO–2-β) → 98B (LUMO-β) (0.98028)
**Ni(2OMe)**$^{-}$ ·H_2O (i)	992.28	0.0025	85A (HOMO–17-α) → 104A (LUMO-α) (0.15019)<br>90A (HOMO–12-α) → 104A (LUMO-α) (0.21188)<br>92A (HOMO–10-α) → 103B (LUMO-β) (-0.12130)<br>95B (HOMO–7-β) → 103B (LUMO-β) (-0.16198)<br>99B (HOMO–3-β) → 103B (LUMO-β) (0.91914)<br>100B (HOMO–2-β) → 103B (LUMO-β) (0.16477)<br>102B (HOMO-β) → 103B (LUMO-β) (0.17365)<br>90A (HOMO–12-α) ← 104A (LUMO-α) (0.12242)<br>99B (HOMO–3-β) ← 103B (LUMO-β) (0.15536)
	886.10	0.1146	99A (HOMO–3-α) → 104A (LUMO-α) (0.12530)<br>103A (SOMO-α) → 104A (LUMO-α) (0.11488)<br>99B (HOMO–3-β) → 103B (LUMO-β) (0.16827)<br>101B (HOMO–1-β) → 103B (LUMO-β) (0.18004)<br>102B (HOMO-β) → 103B (LUMO-β) (0.93845)
	878.13	0.0017	88A (HOMO–14-α) → 104A (LUMO-α) (0.24856)<br>94A (HOMO–8-α) → 104A (LUMO-α) (0.15309)<br>96A (HOMO–6-α) → 104A (LUMO-α) (0.14948)<br>103A (SOMO-α) → 104A (LUMO-α) (0.83202)<br>91B (HOMO–11-β) → 103B (LUMO-β) (0.12779)<br>99B (HOMO–3-β) → 104B (LUMO+1-β) (0.39331)<br>102B (HOMO-β) → 103B (LUMO-β) (0.12811)
	777.64	0.0398	99B (HOMO–3-β) → 103B (LUMO-β) (0.15029)<br>100B (HOMO–2-β) → 103B (LUMO-β) (0.94658)<br>101B (HOMO–1-β) → 103B (LUMO-β) (0.20295)
**Ni(2OMe)**$^{-}$ ·H_2O (i) – H_2O	884.54	0.0019	84A (HOMO–13-α) → 99A (LUMO-α) (0.24408)<br>89A (HOMO–8-α) → 99A (LUMO-α) (0.15555)<br>91A (HOMO–6-α) → 99A (LUMO-α) (0.14618)

			98A (SOMO-α) → 99A (LUMO-α) (0.84543)<br>86B (HOMO–11-β) → 98B (LUMO-β) (0.1287)<br>94B (HOMO–3-β) → 99 B (LUMO+1-β) (0.37573)<br>97B (HOMO-β) → 98 B (LUMO-β) (0.11293)
	873.60	0.1133	94A (HOMO–3-α) → 99A (LUMO-α) (0.12048)<br>97B (HOMO-β) → 98 B (LUMO-β) (0.97012)
	775.50	0.0461	94A (HOMO–3-α) → 99A (LUMO-α) (0.10045)<br>95B (HOMO–2-β) → 98 B (LUMO-β) (0.97879)
**Ni(2OMe)**$^{-}$ ·H_2O (ii)	973.12	0.0077	85A (HOMO–17-α) → 104A (LUMO-α) (0.15460)<br>91A (HOMO–11-α) α → 104A (LUMO-α) (0.2066)<br>92B (HOMO–10-β) β → 103 B (LUMO-β) (0.11592)<br>95B (HOMO–7-β) → 103 B (LUMO-β) (0.15336)<br>99B (HOMO–3-β) → 103 B (LUMO-β) (0.91947)<br>102B (HOMO-β) → 103 B (LUMO-β) (0.26569)<br>91 A (HOMO–11-α) ← 104A (LUMO-α) (0.11818)<br>99B (HOMO–3-β) ← 103 B (LUMO-β) (0.15552)
	862.90	0.1084	99A (HOMO–3-α) → 104A (LUMO-α) (0.12165)<br>99B (HOMO–3-β) → 103 B (LUMO-β) (0.25289)<br>100B (HOMO–2-β) → 103 B (LUMO-β) (0.11957)<br>101B (HOMO–1-β) → 103 B (LUMO-β) (-0.13361)<br>102B (HOMO-β) → 103 B (LUMO-β) (0.92724)
	832.69	0.0009	93A (HOMO–9-α) → 104A (LUMO-α) (0.20998)<br>98A (HOMO–4-α) → 104A (LUMO-α) (-0.64686)<br>93B (HOMO–9-β) → 104 B (LUMO+1-β) (-0.16541)<br>97B (HOMO–5-β) → 104 B (LUMO+1-β) (0.70312)<br>101B (HOMO–1-β) → 103 B (LUMO-β) (-0.13536)<br>98A (HOMO–4-α) ← 104A (LUMO-α) (LUMO-α) (-0.12662)<br>97B (HOMO–5-β) ← 104 B (LUMO+1-β) (0.13073)
	771.21	0.0450	100B (HOMO–2-β) → 103 B (LUMO-β) (0.95547)<br>101B (HOMO–1-β) → 103 B (LUMO-β) (0.21667)
**Ni(2OMe)**$^{-}$ ·H_2O (ii) – H_2O	875.80	0.1167	94A (HOMO–3-α) → 99A (LUMO-α) (-0.12339)<br>97B (HOMO-β) → 98B (LUMO-β) (0.97727)
	775.23	0.0468	95B (HOMO–2-β) → 98B (LUMO-β) (0.98042)

12. XAS analysis

Ni L-edge XAS spectra were acquired in partial fluorescence yield (PFY) mode using a 25 mm² Silicon Drift Detector (FAST SDD, Amptek, USA). The energy resolution was 0.1 eV. A custom-designed cell featuring a 150 nm thick SiC membrane on a 0.625 mm thick Si substrate (NTT-AT Co., Japan) was employed to isolate the ultra-high vacuum of the measurement chamber from the ambient conditions of the liquid samples. The cell was connected to a Teflon tube to circulate temperature-controlled water from 14 °C to 80 °C with a precision of ±0.2 °C. During data collection, the sample cell was raster-scanned by adjusting the scanning speed to move incident X-rays across the thin SiC membrane area (0.3 mm × 3 mm) to minimize X-ray dose and prevent membrane and/or sample damage. The obtained XAS spectra were baseline-corrected and subsequently normalized to the L_3-edge peak maxima. Further details of the experimental setup are described elsewhere.[S1]

13. Coordinates of optimized structures

Table S3. The geometry of the DFT-optimized structure for **Ni(2OMe)** anion (Figure 3b).

1	C	-6.038	0.613	4.873
2	C	-6.461	-0.705	4.581
3	C	-6.491	-1.141	3.248
4	C	-6.113	-0.291	2.215
5	C	-5.694	1.017	2.495
6	C	-5.662	1.452	3.818
7	S	-5.989	1.165	6.541
8	Ni	-6.679	-0.552	7.718
9	S	-6.944	-1.77	5.888
10	S	-6.483	0.687	9.542
11	C	-6.91	-0.396	10.855
12	C	-7.279	-1.73	10.567
13	S	-7.294	-2.294	8.902
14	C	-6.895	0.045	12.186
15	C	-7.24	-0.815	13.222
16	C	-7.609	-2.14	12.947
17	C	-7.623	-2.58	11.626
18	O	-7.191	-0.276	14.494
19	H	-6.812	-2.148	3.009
20	H	-5.395	1.696	1.706

21	H	-5.338	2.463	4.039
22	H	-6.614	1.065	12.421
23	H	-7.881	-2.826	13.738
24	H	-7.907	-3.604	11.408
25	O	-6.182	-0.824	0.942
26	C	-5.809	-0.001	-0.144
27	H	-6.447	0.889	-0.22
28	H	-4.761	0.318	-0.073
29	H	-5.937	-0.608	-1.041
30	C	-7.531	-1.109	15.583
31	H	-8.566	-1.468	15.513
32	H	-6.86	-1.973	15.661
33	H	-7.427	-0.494	16.477

Table S4. The geometry of DFT-optimized structure for **Ni(2OMe)**$^{-}\cdot H_2O$ (i) (Figure S10a).

1	C	-6.086	0.612	4.875
2	C	-6.517	-0.7	4.573
3	C	-6.522	-1.139	3.244
4	C	-6.108	-0.296	2.219
5	C	-5.68	1.008	2.506
6	C	-5.674	1.445	3.828
7	S	-6.067	1.168	6.542
8	Ni	-6.786	-0.536	7.712
9	S	-7.054	-1.752	5.874
10	S	-6.574	0.699	9.532
11	C	-6.967	-0.389	10.852
12	C	-7.336	-1.724	10.572
13	S	-7.403	-2.278	8.903
14	C	-6.921	0.05	12.183
15	C	-7.234	-0.815	13.225
16	C	-7.601	-2.142	12.956

17	C	-7.648	-2.579	11.635
18	O	-7.156	-0.279	14.495
19	H	-6.85	-2.143	2.999
20	H	-5.353	1.68	1.724
21	H	-5.343	2.453	4.055
22	H	-6.638	1.07	12.414
23	H	-7.848	-2.832	13.752
24	H	-7.932	-3.604	11.422
25	O	-4.95	-4.25	7.249
26	H	-5.407	-3.908	8.03
27	H	-5.268	-3.653	6.557
28	O	-6.153	-0.83	0.947
29	C	-5.734	-0.019	-0.133
30	H	-6.354	0.881	-0.23
31	H	-4.684	0.282	-0.032
32	H	-5.846	-0.629	-1.029
33	C	-7.457	-1.118	15.592
34	H	-8.49	-1.486	15.552
35	H	-6.776	-1.976	15.648
36	H	-7.332	-0.504	16.485

Table S5. The geometry of the DFT-optimized structure for **Ni(2OMe)**$^{-}\cdot H_2O$ (i) – H_2O (Figure S10b).

1	C	-6.086	0.612	4.875
2	C	-6.517	-0.7	4.573
3	C	-6.522	-1.139	3.244
4	C	-6.108	-0.296	2.219
5	C	-5.68	1.008	2.506
6	C	-5.674	1.445	3.828
7	S	-6.067	1.168	6.542
8	Ni	-6.786	-0.536	7.712
9	S	-7.054	-1.752	5.874

10	S	-6.574	0.699	9.532
11	C	-6.967	-0.389	10.852
12	C	-7.336	-1.724	10.572
13	S	-7.403	-2.278	8.903
14	C	-6.921	0.05	12.183
15	C	-7.234	-0.815	13.225
16	C	-7.601	-2.142	12.956
17	C	-7.648	-2.579	11.635
18	O	-7.156	-0.279	14.495
19	H	-6.85	-2.143	2.999
20	H	-5.353	1.68	1.724
21	H	-5.343	2.453	4.055
22	H	-6.638	1.07	12.414
23	H	-7.848	-2.832	13.752
24	H	-7.932	-3.604	11.422
25	O	-6.153	-0.83	0.947
26	C	-5.734	-0.019	-0.133
27	H	-6.354	0.881	-0.23
28	H	-4.684	0.282	-0.032
29	H	-5.846	-0.629	-1.029
30	C	-7.457	-1.118	15.592
31	H	-8.49	-1.486	15.552
32	H	-6.776	-1.976	15.648
33	H	-7.332	-0.504	16.485

Table S6. The geometry of the DFT-optimized structure for **Ni(2OMe)**$^{-}$ $\cdot H_2O$ (ii) (Figure S10c).

1	C	-6.182	0.211	5.092
2	C	-5.069	-0.518	4.61
3	C	-5.027	-0.9	3.261
4	C	-6.07	-0.576	2.403
5	C	-7.176	0.147	2.867

6	C	-7.216	0.532	4.204
7	S	-6.246	0.694	6.78
8	Ni	-4.381	-0.071	7.648
9	S	-3.757	-0.919	5.701
10	S	-4.97	0.838	9.577
11	C	-3.689	0.385	10.685
12	C	-2.603	-0.391	10.216
13	S	-2.557	-0.902	8.535
14	C	-3.723	0.785	12.028
15	C	-2.701	0.428	12.9
16	C	-1.619	-0.34	12.444
17	C	-1.584	-0.739	11.11
18	O	-2.835	0.875	14.2
19	O	-5.939	-1.026	1.093
20	C	-6.881	-0.576	0.131
21	H	-4.171	-1.444	2.876
22	H	-7.997	0.407	2.212
23	H	-8.069	1.093	4.571
24	H	-4.549	1.379	12.402
25	H	-0.813	-0.629	13.105
26	H	-0.75	-1.334	10.753
27	H	-6.558	-0.99	-0.823
28	H	-6.891	0.518	0.072
29	H	-7.891	-0.938	0.355
30	O	-5.431	-3.849	0.724
31	H	-5.306	-4.216	1.604
32	H	-5.537	-2.895	0.876
33	C	-1.823	0.54	15.127
34	H	-1.731	-0.546	15.255
35	H	-0.848	0.947	14.832
36	H	-2.124	0.985	16.076

Table S7. The geometry of the DFT-optimized structure for **Ni(2OMe)**$^{-}\cdot H_2O$ (ii) – H_2O (Figure S10d).

1	C	-1.221	-0.032	-2.605
2	C	0.158	0.217	-2.793
3	C	0.663	0.35	-4.095
4	C	-0.178	0.231	-5.193
5	C	-1.546	-0.012	-5.021
6	C	-2.05	-0.139	-3.729
7	S	-1.865	-0.202	-0.979
8	Ni	-0.129	0.066	0.337
9	S	1.211	0.367	-1.4
10	S	-1.479	-0.163	2.074
11	C	-0.419	-0.074	3.468
12	C	0.969	0.119	3.277
13	S	1.617	0.255	1.649
14	C	-0.928	-0.188	4.769
15	C	-0.083	-0.111	5.87
16	C	1.295	0.083	5.692
17	C	1.802	0.195	4.4
18	O	-0.69	-0.235	7.105
19	O	0.43	0.355	-6.439
20	C	-0.4	0.421	-7.588
21	H	1.716	0.558	-4.252
22	H	-2.215	-0.108	-5.866
23	H	-3.108	-0.327	-3.586
24	H	-1.989	-0.337	4.93
25	H	1.97	0.146	6.534
26	H	2.867	0.344	4.257
27	H	0.268	0.58	-8.434
28	H	-1.106	1.256	-7.517
29	H	-0.954	-0.512	-7.74
30	C	0.123	-0.164	8.258

31	H	0.874	-0.964	8.277
32	H	0.634	0.804	8.337
33	H	-0.548	-0.284	9.109

14. Supplementary references

S1. K. Yamazoe, Y. Higaki, Y. Inutsuka, J. Miyawaki, Y.-T. Cui, A. Takahara, Y. Harada, *Langmuir* **2017**, *33*, 3954–3959.

S2. S. Yamamoto, Y. Senba, T. Tanaka, H. Ohashi, T. Hirono, H. Kimura, M. Fujisawa, J. Miyawaki, A. Harasawa, T. Seike, S. Takahashi, N. Nariyama, T. Matsushita, M. Takeuchi, T. Ohata, Y. Furukawa, K. Takeshita, S. Goto, Y. Harada, S. Shin, H. Kitamura, A. Kakizaki, M. Oshima, I. Matsuda, *J Synchrot. Rad.* **2014**, *21*, 352–365.

S3. S. Yokomori, S. Dekura, T. Fujino, M. Kawamura, T. Ozaki, H. Mori, *J. Mater. Chem. C* **2020**, *8*, 14939–14947.

S4. Gaussian 16, Revision C.03, M. J. Frisch, G. W. Trucks, H. B. Schlegel, G. E. Scuseria, M. A. Robb, J. R. Cheeseman, G. Scalmani, V. Barone, G. A. Petersson, H. Nakatsuji, X. Li, M. Caricato, A. V. Marenich, J. Bloino, B. G. Janesko, R. Gomperts, B. Mennucci, H. P. Hratchian, J. V. Ortiz, A. F. Izmaylov, J. L. Sonnenberg, D. Williams-Young, F. Ding, F. Lipparini, F. Egidi, J. Goings, B. Peng, A. Petrone, T. Henderson, D. Ranasinghe, V. G. Zakrzewski, J. Gao, N. Rega, G. Zheng, W. Liang, M. Hada, M. Ehara, K. Toyota, R. Fukuda, J. Hasegawa, M. Ishida, T. Nakajima, Y. Honda, O. Kitao, H. Nakai, T. Vreven, K. Throssell, J. A. Montgomery Jr., J. E. Peralta, F. Ogliaro, M. J. Bearpark, J. J. Heyd, E. N. Brothers, K. N. Kudin, V. N. Staroverov, T. A. Keith, R. Kobayashi, J. Normand, K. Raghavachari, A. P. Rendell, J. C. Burant, S. S. Iyengar, J. Tomasi, M. Cossi, J. M. Millam, M. Klene, C. Adamo, R. Cammi, J. W. Ochterski, R. L. Martin, K. Morokuma, O. Farkas, J. B. Foresman, D. J. Fox, Gaussian, Inc., Wallingford CT, 2016. https://gaussian.com/.

S5. G. M. Sheldrick, *Acta Cryst*. **2015**, *A71*, 3–8.

S6. O. V. Dolomanov, L. J. Bourhis, R. J. Gildea, J. A. K. Howard, H. Puschmann, *J. Appl. Cryst*. **2009**, *42*, 339–341.

S7. J. Ilavsky, *J. Appl. Crystallogr.* **2012**, *45*, 324–328.

S8. www.sasview.org/.

S9. https://easyspin.org/easyspin/documentation/.
